# Hypoxanthine guanine phosphoribosyl transferases SmHGPRTases functional roles in *Schistosoma mansoni*

**DOI:** 10.3389/fmicb.2022.1064218

**Published:** 2022-12-12

**Authors:** Izabella Cristina Andrade Batista, Sandra Grossi Gava, Naiara Clemente Tavares, Carlos Eduardo Calzavara-Silva, Marina Moraes Mourão

**Affiliations:** ^1^Grupo de Helmintologia e Malacologia Médica, Instituto René Rachou, Fundação Oswaldo Cruz, Belo Horizonte, Brazil; ^2^Grupo de Imunologia Celular e Molecular, Instituto René Rachou, Fundação Oswaldo Cruz, Belo Horizonte, Brazil

**Keywords:** *Schistosoma mansoni*, HGPRTase, RNA interference, functional genomics, host–parasite interaction

## Abstract

**Introduction:**

Extracellular/environmental stimuli trigger cellular responses to allow *Schistosoma* sp. parasites adaptation and decide development and survival fate. In this context, signal transduction involving eukaryotic protein kinases (ePKs) has an essential role in regulatory mechanisms. Functional studies had shown the importance of MAPK pathway for *Schistosoma mansoni* development. In addition, early studies demonstrated that Smp38 MAPK regulates the expression of a large set of genes, among them the hypoxanthine-guanine phosphoribosyl transferase 1 (*SmHGPRTase* 1, Smp_103560), a key enzyme in the purine salvage pathway that is part of a family comprising five different proteins.

**Methods:**

First, the regulation of this gene family by the MAPKs pathways was experimentally verified using Smp38-predicted specific inhibitors. In silico analysis showed significant differences in the predicted structure and the domain sequence among the schistosomal HGPRTase family and their orthologs in humans. In order to interrogate the *HGPRTases* (Smp_103560, Smp_148820, Smp_168500, Smp_312580 and Smp_332640, henceforth SmHGPRTase −1, −2, −3, −4, −5) functional roles, schistosomula, sporocysts, and adult worms were knocked-down using specific dsRNAs.

**Results:**

Our results suggest that SmHGPRTases activity has an essential role in sporocysts and schistosomula development since significant differences in viability, size, and/ or shape were observed after the *in vitro* knockdown. Also, the knockdown of *SmHGPRTases* in schistosomula influenced the ovary development and egg maturation in female adult worms during mammalian infection. We also observed alterations in the movement of female adult worms knocked-down *in vitro*. Most of these results were shown when all gene family members were knocked-down simultaneously, suggesting a redundant function among them.

**Discussion:**

Thus, this study helps to elucidate the functional roles of the *SmHGPRTase* gene family in the *S. mansoni* life cycle and provides knowledge for future studies required for schistosomiasis treatment and control.

## Introduction

Schistosomiasis is a neglected tropical disease caused by trematode worms from the *Schistosoma* genus and is considered a major health and economic problem in developing countries. The transmission is related to precarious water treatment and sanitation conditions, combined with the presence of the snail vector (World Health Organization, 2018). *Schistosoma mansoni* is one of the main species causatives of schistosomiasis and can survive in the mammalian host for years or decades (Pearce and Sher, 1987). According to the World Health Organization, 78 countries reported transmission of this disease, and at least 241.3 million people required preventive chemotherapy with praziquantel (PZQ) in 2020 (World Health Organization, 2021).

To date, PZQ is the only treatment available for schistosomiasis. However, although this drug is effective against all species of *Schistosoma*, there are limitations to its use, including the development of resistance (Botros and Bennett, 2007; Doenhoff et al., 2008; Greenberg, 2013). Thereat, the search for new drug targets to support schistosomiasis treatment is extremely important.

The study of molecular interactions between hosts and parasite is essential to understanding this parasitic infection, its adaptation within the hosts, and pathogenesis (Cuesta-Astroz et al., 2019). Extracellular stimuli trigger cellular responses to allow the development and survival of parasites of the genus *Schistosoma*. Protein Kinases (PKs) play a major role in mediating these signals, which involve integrated networks that interact mostly by switching proteins activity status, performing essential functions in cell control (Hanks et al., 1988; Andrade et al., 2011). Despite the availability of many options that would allow the employment of piggyback strategies, specific targets regulated by those kinases could also be handy in order to avoid side effects (Boyle and Koleske, 2007; Eglen and Reisine, 2009).

Recent studies showed that the Smp38 MAPK signaling pathway is essential for the development, reproduction, and survival of *S. mansoni* (Avelar et al., 2019). Thus, despite being a promising target against the parasite, inhibition of hosts enzymes could be a concern and a barrier to be circumvented during drug development. Previous functional studies of the Smp38 gene have shown that this pathway regulates the expression of a large set of genes in *S. mansoni*, including the hypoxanthine-guanine phosphoribosyltransferase (*SmHGPRTase* 1; Avelar et al., 2019; Gava et al., 2019), an important protein in parasite biology, that has been extensively interrogated as a drug target (Senft and Crabtree, 1983; Dovey et al., 1984; Pereira et al., 2008; Romanello et al., 2019). This enzyme is included in a family of enzymes comprised by five different proteins whose role is to convert purine bases, hypoxanthine and guanine, to their respective nucleotides, inosine monophosphate (IMP) and guanosine monophosphate (GMP), in the presence of 5-phosphorylribose 1-pyrophosphate (PRPP; Senft and Crabtree, 1983). Studies demonstrated that *S. mansoni* adult worms were unable to incorporate ^14^C-glycine into a purine ring, evidencing the dependence of an external supply of preformed bases for nucleotide synthesis and the absence of the *de novo* purine biosynthesis pathway (Miech et al., 1975). Since *S. mansoni* depends entirely on the salvage pathway to generate purines, enzymes like the SmHGPRTase family, involved in this pathway, could be critical to the parasite’s life cycle and because of that many studies have shown that HGPRTases, regulated by PKs, are promising targets for the development of new drugs (Senft and Crabtree, 1983; Dovey et al., 1984). Example of that is the acyclic nucleoside phosphonates, which have previously been shown to be potent inhibitors of *Plasmodium falciparum* HGPRTase, while showing excellent selectivity for the parasite when compared with the human enzyme (Kaiser et al., 2017; Keough et al., 2018). Substructures of phosphoribosyl pyrophosphate (PRPP), an original substrate of HGPRT, are also being extensively studied for that and structure-based virtual screening and computational study had shown that these inhibitors can be potential drugs for *Trypanosoma cruzi* (Vidhya and Ponnuraj, 2021).

In the present report, we interrogate the functional roles of a gene family regulated by the MAP kinase pathway in *S. mansoni*, *SmHGPRTases*, in different stages of the parasite’s life cycle and suggest that those proteins could be parasite-specific druggable targets.

## Materials and methods

### Target genes

In this study we have focused on the characterization of the hypoxanthine-guanine phosphoribosyl transferase (*SmHGPRTase*) gene family, regulated by Smp38 MAPK pathway. *S. mansoni* presents five different genes encoding SmHGPRTases, namely Smp_103560 (comprising two isoforms Smp_103560.1 and Smp_103560.2), Smp_148820, Smp_168500, Smp_312580 and Smp_332640; henceforth referred as *SmHGPRTase* 1, *SmHGPRTase* 2, *SmHGPRTase* 3, *SmHGPRTase* 4, and *SmHGPRTase* 5, respectively. According to the *S. mansoni* genome (v. 7) deposited in the WormBase Parasite database (Howe et al., 2017).

### Parasites

All the experiments using animals were reviewed and approved by the Ethics Commission on Animal Use from Fundação Oswaldo Cruz under license numbers LW12/16 for hamsters and LM05/18 for mice.

The *S. mansoni* LE cercaria strain was acquired from the Mollusk rearing facility “Lobato Paraense” of René Rachou Institute – FIOCRUZ using *Biomphalaria glabrata* as the intermediate snail host.

To obtain *S. mansoni* adult worms and sporocysts, six-week-old female Golden hamsters (*Mesocricetus auratus*) were infected subcutaneously with 400 cercariae each. Forty-two days after infection, animals were anesthetized with xylazine hydrochloride (10 mg/kg; Syntec) and ketamine hydrochloride (150 mg/kg; Syntec), followed by overdose euthanasia with 2.5% sodium thiopental (150 mg/kg; Cristália). Then, perfusion was performed (Pellegrino and Siqueira, 1956; Tavares and Mourão, 2021) to recover adult worms, eggs were recovered from hamsters’ livers and sporocysts obtained as described by Mourão et al. (2009). Schistosomula were obtained by mechanical transformation of the cercariae (Milligan and Jolly, 2011).

### Analysis of *SmHGPRTase* regulation by Smp38

According to Gava et al. (2019), the *SmHGPRTase 1* expression is down-regulated in Smp38 knocked-down schistosomula. To experimentally verify that regulation, an inhibitor previously identified by our group to bind in the ATP binding site of Smp38 was used (inhibitor NCC – 00001994 from the Managed Chemical Compound Collection – MCCC; Moreira et al., 2022) The inhibitor NCC – 00001994 was used at a final concentration of 10 μM in cultures containing 5,000 schistosomula in 1 ml of Glasgow Minimum Essential Medium (GMEM; Sigma-Aldrich) supplemented with 20 mM HEPES (Sigma-Aldrich), 0.1% lactalbumin hydrolysate (Vetec), 0.1% D-glucose (Sigma-Aldrich), 0.5 μM hypoxanthine (Sigma-Aldrich), 1 μM hydrocortisone (Sigma-Aldrich), 0.5% MEM vitamin solution (Gibco), 5% Schneider (Gibco), 1% penicillin and streptomycin (Gibco), and 2% heat-inactivated fetal bovine serum (FBS; Gibco). Parasites cultured with 0.2% of dimethyl sulfoxide (DMSO; negative control) were used as controls. To verify morphological alterations and viability, parasites were incubated for 30 h at 37°C, 5% CO_2_, and 95% humidity, prior to the phenotypic assessment by inverted microscope (ABO 100 – ZEISS). Viability assessment was performed by the addition of 5 μg/ml of propidium iodide and visualization under a fluorescent inverted microscope (ABO 100 - ZEISS) using a 544 nm wavelength. After inhibitor exposure, the RNA was extracted, the cDNA was synthesized and the gene expression was analyzed by qPCR, as further detailed.

### Protein modeling and analysis

The sequences of *S. mansoni* proteins used in this study were obtained from their predicted coding sequences in the *S. mansoni* genome (v. 7) deposited in the WormBase Parasite database^1^ (Howe et al., 2017) and the sequence for human protein was obtained from the Uniprot database^2^ (PDB ID P00492; Bateman et al., 2021). Protein domains (PF00156) coordinates were retrieved from the Pfam^3^ (El-Gebali et al., 2019) and comparisons between *S. mansoni* and human sequences were performed using the MAFFT alignment program^4^ (Katoh et al., 2019). The identity percentage between domains’ sequences was calculated by pairwise comparisons using the Jalview software^5^ (Waterhouse et al., 2009).

SmHGPRTase 1, SmHGPRTase 2, SmHGPRTase 3, SmHGPRTase 4, and SmHGPRTase 5 tridimensional modeling were carried out to compare the structures of *S. mansoni* proteins and the corresponding human orthologs. As SmHGPRTases 4 and 5 seems to be a duplication *in tandem* and present the same sequence, the *in silico* analysis for these two proteins was performed only once (SmHGPRTases 4/5). The proteins were first modeled using the Phyre2 web portal^6^ (Kelley et al., 2015). After modeling, the predicted structures were aligned, and a subsequent comparison was carried out using the Chimera 1.13.1 program (Pettersen et al., 2004).

### Single cell RNA-seq (scRNAseq) analysis

To verify the *SmHGPRTases* expression in the different cell types of adult *S. mansoni* worms, single-cell RNAseq (scRNAseq) data were obtained from the Gene Expression Omnibus database (GEO^7^, BioProject PRJNA611783, SRASRP252217; Wendt et al., 2020). The RDS file containing the expression data in the different cell types was loaded in the R software (v4.1.2) (R Core Team, 2021) using the Seurat package (v4.1.1) (Satija et al., 2015) and used to build a heatmap with the package ComplexHeatmap (v2.10.0) (Gu et al., 2016).

### Double-stranded RNA (dsRNA) synthesis

To functionally assess the SmHGPRTases proteins, specific primers containing the T7 promoter sequence were designed based on their nucleotide sequences in the *S. mansoni* genome (v. 7) available in the WormBase Parasite database and used in a PCR to amplify fragments ranging from 250 to 578 bp. The unspecific control, green fluorescent protein (GFP), was synthesized from a fragment cloned into a pCRII plasmid (AddGene). PCR products were analyzed in 1% agarose gel and purified using QIAquick Gel Extraction Kit (Qiagen), following the supplier’s protocol. PCR products were previously cloned into pGEM-T Easy vector (Promega) and sequenced by Sanger sequencing using specific primers (Supplementary Table S1). After sequence confirmation, PCR products were used for double-stranded RNA (dsRNA) synthesis. The dsRNAs were synthesized using the T7 RiboMAX Express RNAi System kit (Promega) according to the manufacturer’s protocol. As the *SmHGPRTase 2* sequence is similar to *SmHGPRTases 4 and 5*, we used only one dsRNA targeting the three genes, thus, the results for these genes are represented as *SmHGPRtase 2/4/5* hereafter.

### Ds-RNA exposure and phenotypic assessment in different life stages of *Schistosoma mansoni*

#### Sporocysts

The sporocysts (20,000/well) were maintained in 6-well polystyrene tissue culture plates with Chernin’s balanced saline solution (CBSS) supplemented with 1 g/L glucose (Vetec), 1 g/L trehalose, and 1% penicillin/streptomycin (Gibco) at 28°C. In each well were added 50 nM of dsRNA (*SmHGPRT 1*, *SmHGPRT 2/4/5*, *SmHGPRT 3*, or *GFP*; Mourão et al., 2009). The parasites were also exposed to a combination of the dsRNAs targeting the five *SmHGPRTases* – termed as “combined group.” This group consisted of 50 nM of each dsRNA of the *SmHGPRTases*, and 150 nM of GFP-dsRNA was used for the respective nonspecific control. Parasites not exposed to dsRNAs were included as an “untreated control.” Parasite cultures were observed daily using an inverted microscope (ABO 100 – ZEISS) to verify phenotypic changes. The viability was assessed with the addition of 5 μg/ml of propidium iodide under a fluorescent inverted microscope (ABO 100 – ZEISS) using a 544 nm wavelength. Parasite images were recorded using the Axion Vision REL 4 software (ZEISS) for 10 days. The area (μm^2^) of each sporocyst was measured using AxioVision 4.8 software. The experiments were performed in three independent biological replicates.

#### Schistosomula

Schistosomula cultures (30,000/well) were maintained in 6-well polystyrene tissue culture plates in 3 ml GMEM supplemented as previously mentioned in the inhibitor exposure section. After cercariae transformation, schistosomula were exposed to 100 nM of dsRNAs (*SmHGPRT 1*, *SmHGPRT 2/4/5*, *SmHGPRT 3*, or *GFP*; Andrade et al., 2011). DsRNAs at 200 nM (~70 nM of each dsRNA) were added to the “combined” and the unspecific controls. An untreated control was also included. Cultures were incubated at 37°C, 5% CO_2_, and 95% humidity and were daily observed under a fluorescent inverted microscope (ABO 100 – ZEISS) to verify the viability as described previously for sporocysts. The experiments were performed in three independent biological replicates.

#### Adult worms

Adult worms recovered by perfusion were washed three times with Roswell Park Memorial Institute medium 1640 (RPMI) supplemented with 1% penicillin and streptomycin (Gibco). Worm pairs were manually separated, washed, and electroporated with 25 μg of each specific dsRNA (*SmHGPRT 1*, *SmHGPRT 2/4/5*, *SmHGPRT 3*, or *GFP*) using 4 mm cuvettes (Bio-Rad) at 125 V for 20 ms (Gava et al., 2019). For the combined group, were added 8.5 μg of each dsRNA (~25 μg total). An untreated control was also evaluated. Eight worm pairs were transferred to 6-well polystyrene tissue culture plates and maintained in RPMI 1640 medium supplemented with 2% penicillin and streptomycin (Gibco) and 10% FBS (Gibco) and incubated at 37°C, 5% CO_2_, and 95% humidity in a CO_2_ incubator.

Worm motility was assessed using 24-well polystyrene culture plates containing eight male or eight female worms per well, maintained as mentioned above. The worm’s movement was recorded for 90 s using WormAssay software (Marcellino et al., 2012) for 10 days. The experiments were performed in three biological replicates.

### RNA extraction, cDNA synthesis, and real-time quantitative PCR (qPCR) analysis

After dsRNA exposure, RNA extractions using the TRIzol Reagent (Invitrogen) were performed using 5,000 parasites on days 2, 4, and 7, for sporocysts, and days 2, 3, and 7 for schistosomula (Tavares et al., 2020). Differently, two pairs of worms on days 2, 4, and 7 were macerated with TRIzol Reagent (Invitrogen), and the RNA extractions were carried out associated with the SV Total RNA Isolation System (Promega), as described previously (Tavares et al., 2020). All RNA samples were treated with Turbo DNase (Ambion). The total RNA was quantified using Qubit RNA HS Assay Kit (Invitrogen) at a Qubit 2.0 Fluorometer (Invitrogen) and stored at −70°C.

To assess transcript knockdown on parasites exposed to dsRNAs, the cDNA was synthesized using ImProm-II^™^ Reverse Transcription System (Promega). Primers for qPCR were designed using the Primer 3 software^8^ to amplify fragments ranging from 70 to 150 bp (Supplementary Table S1). qPCR was performed using GoTaq^®^ qPCR Master Mix (Promega) on a ViiA 7 Real-Time PCR System (Thermo Scientific). The samples were normalized using the Cytochrome C oxidase I (Sm*cox*I, Smp_900000) and the actin-related protein 10 (Sm*arp*10, Smp_093230) expression levels geometric mean and all samples were assessed in three technical replicates. The relative expression was analyzed by comparing the expression levels of each gene to those from unspecific and untreated control groups (Livak and Schmittgen, 2001).

To investigate the mRNA expression profile of each target gene throughout different parasite’s life stages, we also used the above protocol and relative qPCR analysis. In this evaluation, the transcription levels of each gene in miracidia, sporocysts, cercariae, schistosomula, adult males, and adult females were normalized using the geometric mean transcription rate of three reference genes: Sm*cox*I, Sm*arp*10, and SmFAD-dependent oxidoreductase (Sm*fad*, Smp_089880).

### High-performance liquid chromatography

To check whether adenosine levels were decreased after *SmHGPRTases*-knockdown, a High-Performance Liquid Chromatography (HPLC) method was performed to separate and quantify the adenosine content in the samples. First, a calibration curve of the adenosine at seven different concentrations (1,000, 500, 250; 125; 62.5; 31.25; 15.625 ng/ml) was generated. To perform the experiment, 150 μl of methanol containing 100 ng/ml of phenacetin (Sigma-Aldrich) were added to the reactions from the curve and samples. Following, parasites (four adult male worms, four adult female worms, ~3,000 sporocysts, and ~ 3,000 schistosomula) from unspecific control, and parasites treated with *SmHGPRTase 1*, *SmHGPRTase 2/4/5*, and *SmHGPRTase 3* -dsRNAs, separately or in combination, were macerated and centrifuged at 13,000 rpm for 10 min. The supernatant was transferred to a tube containing C18 resins (Sigma-Aldrich) and centrifuged. Then, the supernatant recovered from the resin was lyophilized in a Vacuum Concentrator (Eppendorf) for 30 min, then resuspended in 50 μl of Milli-Q water and transferred to LC–MS tubes (Sigma-Aldrich). The HPLC was performed on a Nexera UHPLC (Shimadzu) hyphenated system on a maXis ETD high-resolution ESI-QTOF mass spectrometer (Bruker) controlled by the Compass 1.5 software (Bruker). The 20 μl volume of the metabolite extracts were injected into a column Shim-Pack XR-ODS-III (C18, 2.2 μm, 2.0 × 150 mm – Shimadzu) at 30°C in a flow of 400 μl/min. The adenosine detection was based on the exact mass and retention time obtained from the standard calibration curve. Individual variations in extraction efficiency were normalized using the internal phenacetin standard. Adenosine detection and quantification were performed using the QuantAnalysis program from the Compass software. The experiments were performed in three biological replicates.

### *In vivo* experiments

Knocked-down schistosomula exposed to the three *SmHGPRTases*-dsRNAs, which included all five enzymes in combination, were used for *in vivo* experiments. As mentioned before, schistosomula were also exposed to an unspecific dsRNAs. After 3 days of dsRNA exposure and transcript levels confirmation by RT-qPCR, 350 parasites from each experimental group, were subcutaneously inoculated in six-week-old female Swiss mice (*Mus musculus*). Forty-two days after infection, animals were euthanized by overdose as described before. Six mice were infected in each experimental group and three independent biological replicates were performed.

After adult worms’ recovery by perfusion, they were counted, fixed in Alcohol-Formalin-Acetic Acid (AFA) and stained with chloride carmine (Machado-Silva et al., 1997). Then, confocal images were captured using an inverted microscope Eclipse Ti-E (Nikon) with Confocal C2 plus (Nikon) at 546 and 488 nm wavelengths. Confocal images of 12 to 15 female and 12 male worms from knocked-down parasites recovered from mice were analyzed. The ovary area and tubercles’ height were measured and analyzed using the NIS-Elements software (Nikon).

Additionally, mice’s livers and intestines were removed, weighed, and incubated with 10% KOH for egg recovery and quantification (Tavares and Mourão, 2021). The mice ileum was pressed with microscope slides to evaluate the egg maturation (stages 1, 2, 3, and 4; Mati and Melo, 2013).

### Statistical analysis

First, a normality test using Shapiro–Wilk was performed for RT-qPCR data, adenosine levels assessment, egg maturation, and morphometric analyses. Then, the significant differences compared to control conditions were analyzed by t-test. For the parasite area, the normality test was performed using D’Agostino-Pearson, followed by *t*-test. For mortality rate and movement in adult worms, two-way analysis of variance (ANOVA) and Sidak as the *post hoc* test were used. Statistical significance was defined as *p* ≤ 0.05. All graphics and statistical analyses were performed using GraphPad Prism 8 (La Jolla, CA, United States).^9^

## Results

### Smp38 MAPK pathway regulates *SmHGPRTases* expression

**Figure 1:**
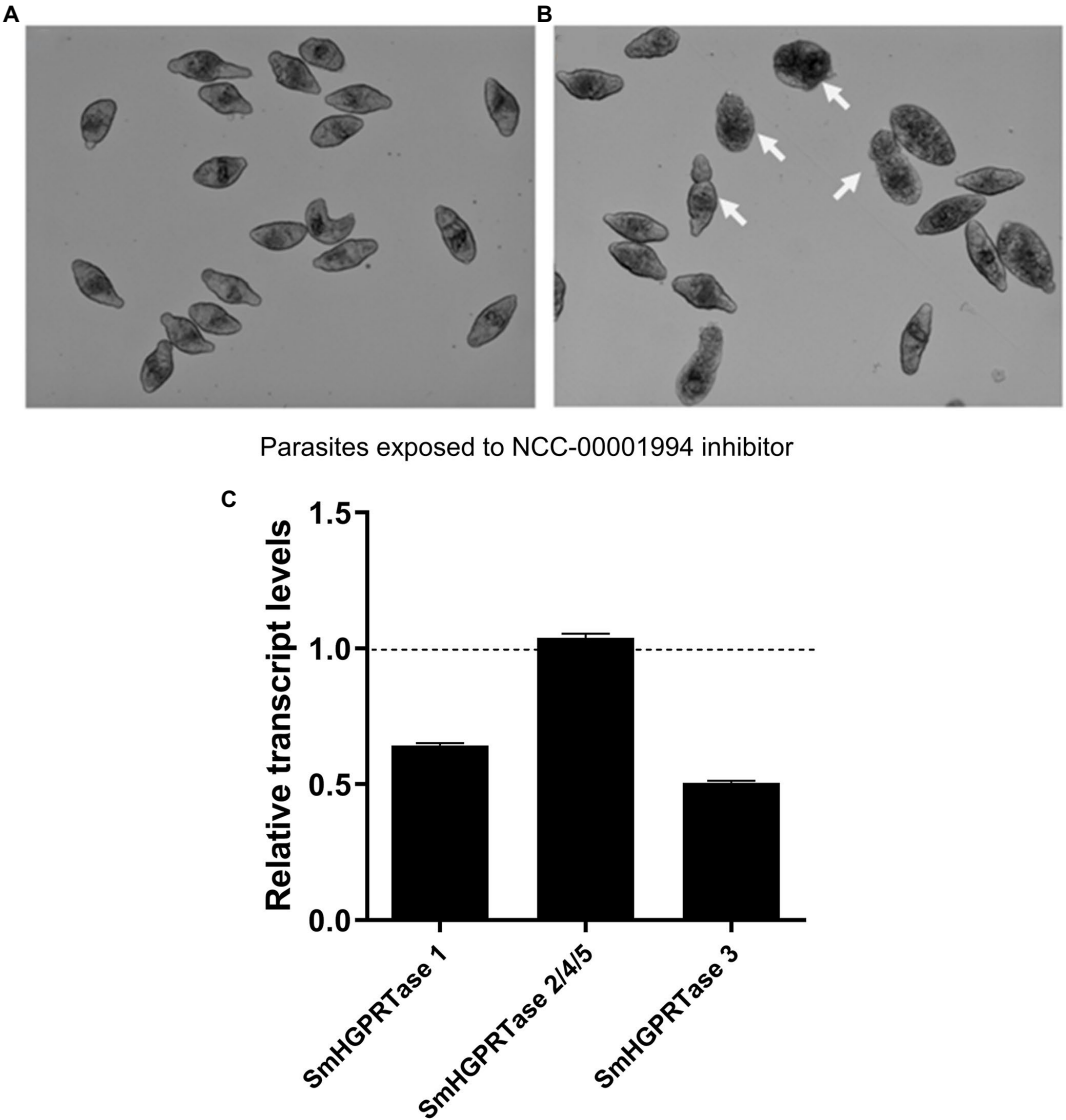
Experimental corroboration of Smp38 MAP kinase pathway in the regulation of the expression of *SmHGPRTase 1, 2/4/5,* and *3*. Representative images of schistosomula from the control **(A)** and schistosomula exposed to Smp38 predicted inhibitor NCC-00001994. **(B)** Arrows point to morphological changes in the parasites. **(C)** Bars representing the *SmHGPRTase 1, 2/4/5, 3* transcript levels in schistosomula exposed to Smp38 predicted inhibitor NCC-00001994. Data were normalized relative to the *SmHGPRTases* expression levels in parasites exposed to DMSO (dashed line).

After the *in silico* analysis, we sought to confirm whether *SmHGPRTases* are regulated by the Smp38 MAPK pathway as previously described (Gava et al., 2019). Thus, schistosomula were exposed to Smp38 (NCC–00001994) predicted inhibitor (Moreira et al., 2022). After 30 h of culture, as described by Moreira et al. (2022) it was observed alterations in schistosomula phenotypes, like the presence of a dark middle-region and round bodies (Figures 1A,B). Additionally, the transcript levels of *SmHGPRTase 1*, *2/4/5,* and *3* were evaluated after the parasite exposure to the inhibitor. When the Smp38 predicted inhibitor was used, the *SmHGPRTase 1* and *3* transcript levels were decreased by 37 and 50% respectively, compared to parasites exposed to the vehicle control (DMSO 0.2%; Figure 1C). *SmHGPRTases 2/4/5* transcript levels were not altered after exposure to the predicted Smp38 inhibitor.

### *SmHGPRTases* are different from their human orthologs

First of all, we aimed to comparing the *S. mansoni* SmHGPRTases target of this study to the human orthologs. Thus, an *in silico* analysis was performed comparing the five SmHGPRTases proteins identified in *S. mansoni* with their respective human counterparts.

The characteristic domain of an HGPRTase is the Phosphoribosyl transferase domain (Family Pribosyltran – PF00156), which catalyzes the substitution of the 5-phospho-a-D-ribose-1-diphosphate group 1-pyrophosphate (PRPP) with a purine base (adenine, guanine, hypoxanthine, or xanthine) to form the corresponding nucleoside in the nucleoside-5-monophosphate form (El-Gebali et al., 2019). The percentage of identity between the Phosphoribosyl transferase domain sequences from *S. mansoni* and their human ortholog counterpart is 50.61% for SmHGPRTase 1, 35.23% for SmHGPRTase 2, 40.49% for SmHGPRTase 3 and 36.87% for SmHGPRTases 4 and 5. The sequence position of each domain is represented in Supplementary Table S2.

**Figure 2:**
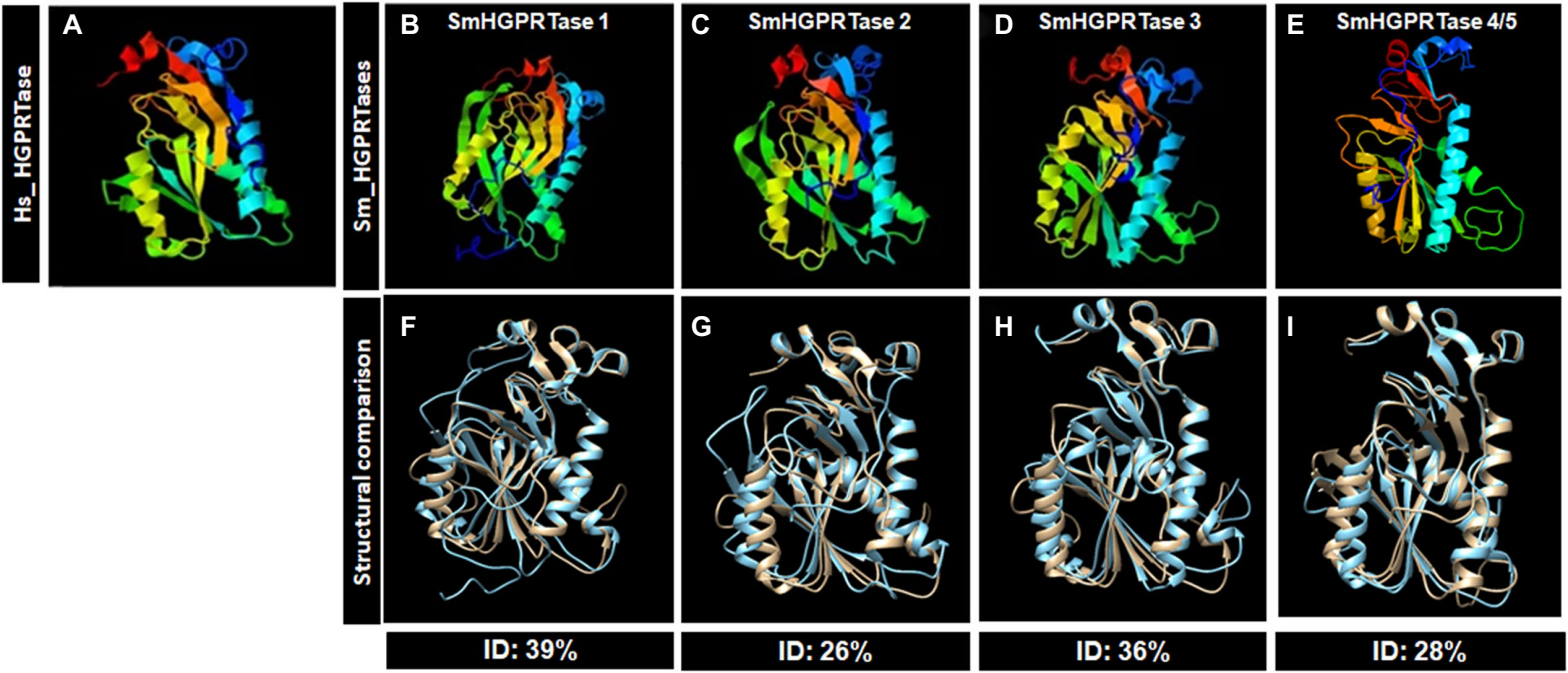
Predicted tridimensional structures for HGPRTases from *Schistosoma mansoni* and their human ortholog. Using the Phyre2 web portal the HGPRTase proteins from human **(A)** and *S. mansoni* (SmHGPRTase 1 – **B**, SmHGPRTase 2 – **C**, SmHGPRTase 3 – **D**, and SmHGPRTase 4/5 – **E**) were modeled and overlapped. Secondary structure elements are rainbow-colored from blue at the N-terminus to red at the C-terminus **(A–E)**. The percentage identities were calculated using the predicted models and the overlaps between the respective structures are represented below **(F–I)**. In gray, human protein; In blue, *Schistosoma* proteins.

By overlapping the predicted tridimensional structures of each HGPRTase protein, SmHGPRTase 1 presented the greatest identity with the human protein (39%; Figures 2A,B,F). Considering the domain sequences, the proteins with lower identity, SmHGPRTase 2 and SmHGPRTase 4/5, also showed low similarity when comparing the tridimensional structures (around 27%; Figures 2A,C,E,G,I), while SmHGPRTase 3 presented 36% identity with the human protein the tridimensional structures were compared (Figures 2A,D,H).

### Expression profile of *SmHGPRTases* in different cell types of *Schistosoma mansoni*

**Figure 3:**
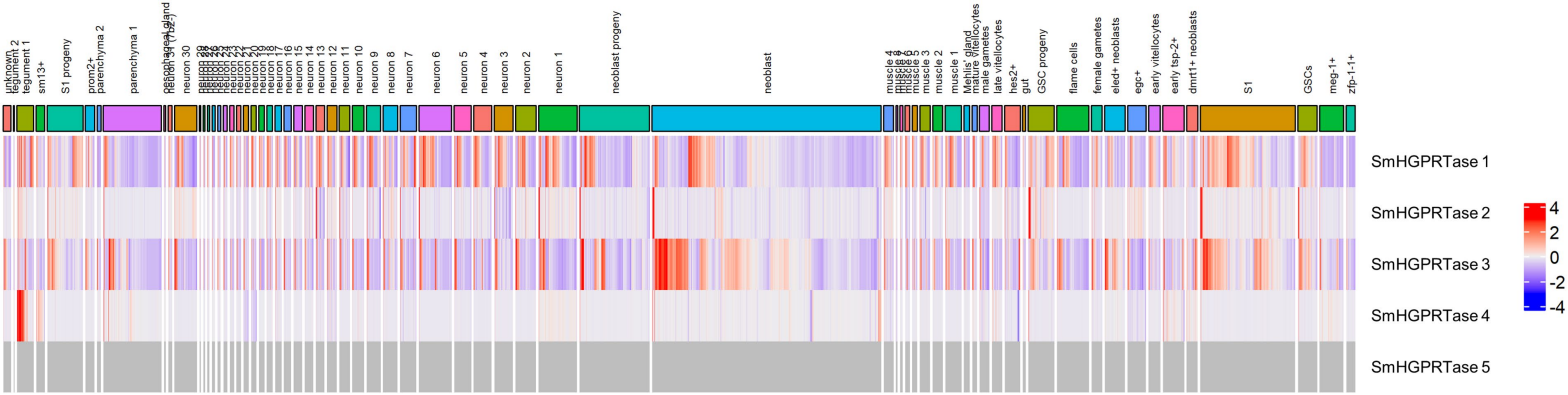
*SmHGPRTAses* expression patterns in different cell types observed in *Schistosoma mansoni* adult worms. The columns indicate the different cell types identified in adult worms and the lines represent the different *SmHGPRTAses* analyzed. The color scale indicates higher (red) or lower (blue) expression of transcripts. The undetectable expression is represented in gray.

First, an overall assessment of *SmHGPRTases* expression in the different cell lines was performed. The analysis of the scRNAseq data provided by Wendt et al. (2020) allowed the verification of the *S. mansoni SmHGPRTAses* expression in different cell types of *S. mansoni* adult worms (Figure 3). *SmHGPRtases 1* and *3* are highly expressed in almost all cell types, while *SmHGPRtase 2* is less expressed although in diverse cell types, and *SmHGPRtase 4* is more expressed in clusters of cells from the tegument (Supplementary Figure S1).

### Expression profile of *SmHGPRTases* in different *Schistosoma mansoni* life stages

Once *SmHGPRTases 1* and *3* were experimentally proven to be regulated by Smp38 MAPK pathways and are distinct from their human counterpart, we hypothesized they could be promising candidates for further functional characterization. First, we sought, to investigate the expression profiles of each target gene throughout different *S. mansoni* life stages, the *SmHGPRTases* transcript levels were assessed in miracidia, sporocysts, cercariae, schistosomula, and male and female adult worms.

**Figure 4:**
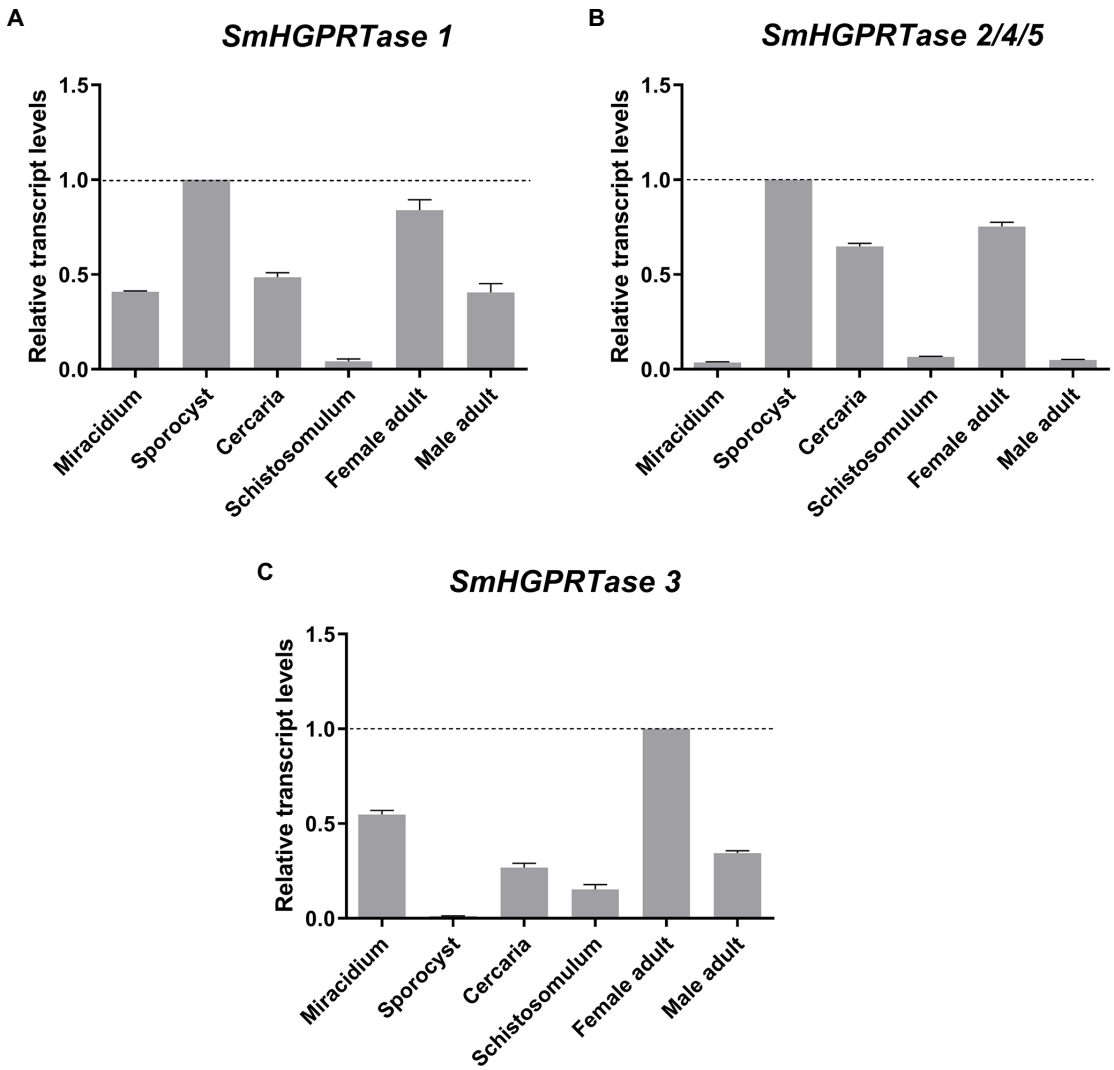
Expression profile of *SmHGPRTases* throughout different parasite’s life stages. Bars showing the relative transcript levels of *SmHGPRTase 1* **(A)**, *SmHGPRTase 2/4/5* **(B)**, and *SmHGPRTase 3* **(C)** in the different *S. mansoni* life stages: miracidium, sporocyst, cercaria, schistosomulum, male, and female adult worm. **(A,B)** Data were normalized relative to the sporocyst stage (dashed line). **(C)** Data were normalized relative to the female adult worm stage (dashed line).

*SmHGPRTase 1* exhibited the highest expression levels in sporocysts, followed by female adult worms, and the lowest levels in schistosomula. Miracidia, male adult worms, and cercariae presented approximately half of the amount exhibited by sporocysts (Figure 4A). *SmHGPRTases 2/4/5* also showed the highest expression levels in sporocysts and the second-highest expression levels in the female adult worm, followed by cercariae. Schistosomula and male adult worms presented low expression levels and miracidia presented the lowest levels (33 times less expressed than in sporocysts; Figure 4B). For *SmHGPRTase 3*, the highest expression levels were presented by female adult worms, which was twice the amount presented by miracidia which exhibited the second highest expression levels. Sporocysts presented the lowest expression levels (Figure 4C).

### *SmHGPRTase* knockdown reduces adenosine levels in sporocysts and influences their viability

Once sporocysts presented the largest transcriptional levels of four *SmHGPRTases* (1 and *2/4/5*) we sought to evaluate the predicted functional roles of *SmHGPRTases*. The genes were knocked-down in sporocysts by exposure to specific dsRNAs for each gene or in combination by targeting the five *SmHGPRTases*.

**Figure 5:**
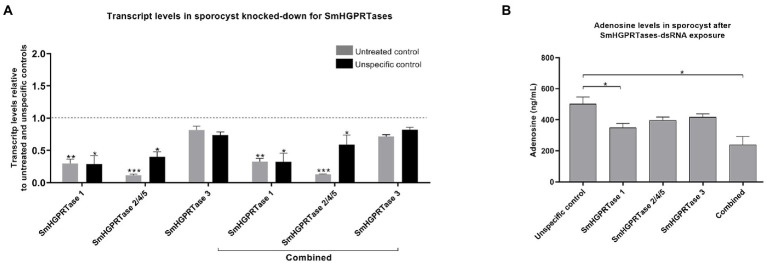
*SmHGPRTases* transcript levels after sporocysts exposure to dsRNAs and knockdown effects in adenosine levels. **(A)** Bars representing the transcript levels of *SmHGPRTase 1, SmHGPRTase 2/4/5,* and *SmHGPRTase 3* in sporocysts after exposure to the specific dsRNAs separately or in combination (combined) relative to the untreated (gray) or unspecific control (black) after 4 days. Above the bars are represented the standard error of the mean of three replicates. The dashed line represents the normalized values in the controls. **(B)** Bars representing the adenosine levels in sporocysts in the respective groups: unspecific control, parasites treated with *SmHGPRTase 1*-dsRNA, *SmHGPRTase 2/4/5*-dsRNA, *SmHGPRTase 3*-dsRNA, and the combined group. Above the bars are represented the standard error of the mean of three replicates. After verifying the normality using the Shapiro–Wilk test, significant differences compared to control conditions were analyzed by unpaired *t*-test (*p ≤ 0.05; **p ≤ 0.01; ***p ≤ 0.001).

After knockdown, *SmHGPRTase 1* transcript levels were periodically evaluated in sporocysts (Figure 5A; Supplementary Figure S2) and we found that the lowest levels of transcript were achieved on the fourth day after dsRNA exposure, reducing by 71% regarding the untreated and unspecific controls. For *SmHGPRTase 2/4/5*, the highest transcript knockdown was achieved after 7 days of sporocysts exposure compared to the untreated and unspecific control groups, resulting in 77–89% reduction. In turn, *SmHGPRTase 3* transcript levels did not reduce after 2 days of dsRNA exposure and presented a reduction of 22–35% after 7 days of dsRNA exposure when compared to the controls. Similar knockdown effects were observed when we used a dsRNA combination targeting the five *SmHGPRTases,* likewise the best knockdown effect was detected on the fourth day of dsRNA exposure 68% for *SmHGPRTase 1,* 88–41% for *SmHGPRTase 2/4/5,* and 25% for *SmHGPRTase 3* in comparison to controls.

Once we determined the days when the transcript levels were lower after *SmHGPRTases* knockdown in sporocysts, biochemical tests were performed to check whether adenosine levels were also decreased, since these enzymes are supposed to be involved in the purine salvage pathway. We observed that the adenosine levels were 30% lower in the *SmHGPRTase 1* knocked-down sporocysts compared with the unspecific control. For the *SmHGPRTase* combined group, a 50% decrease in adenosine levels was observed when compared with the unspecific control after 4 days of dsRNA exposure (Figure 5B).

**Figure 6:**
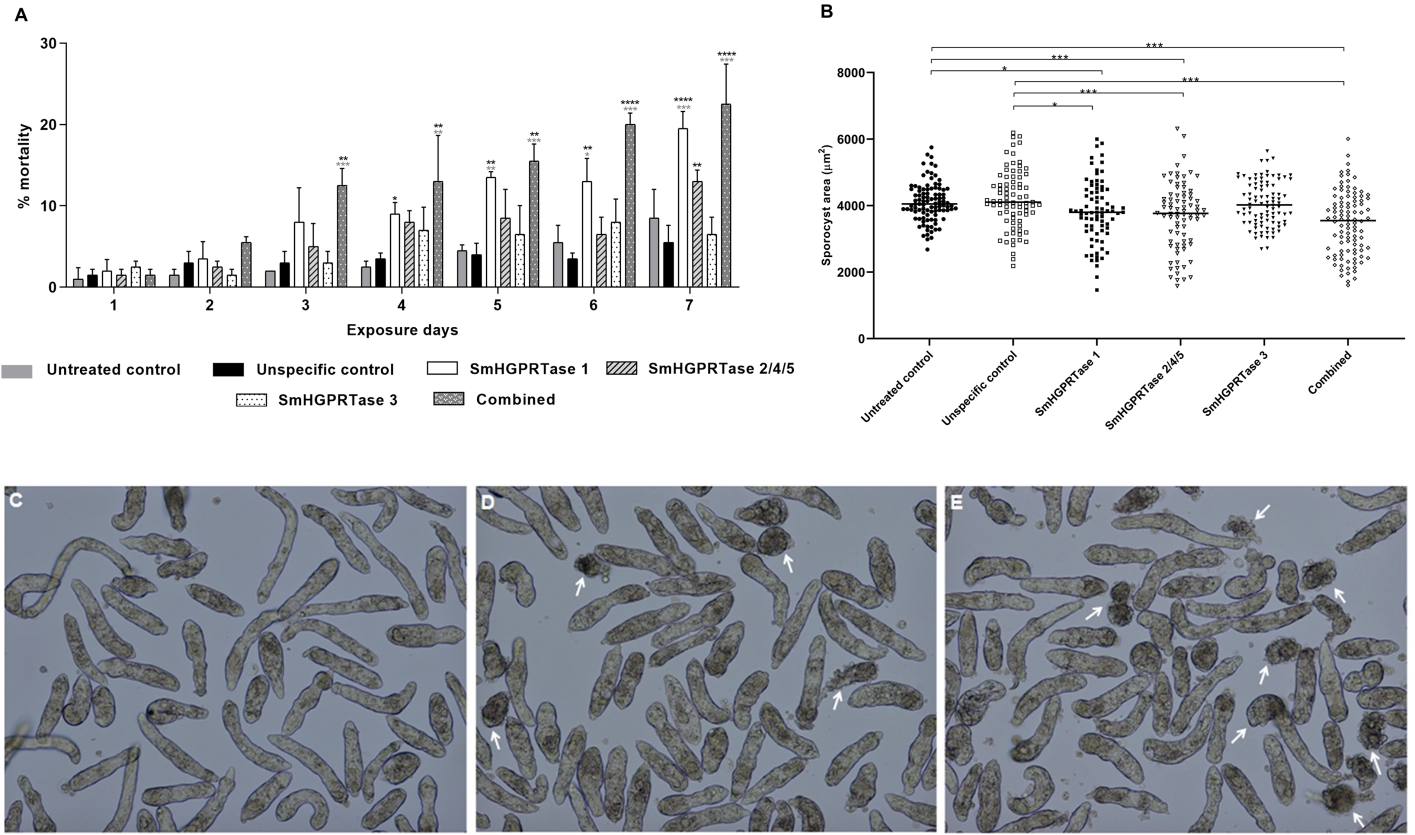
Viability of sporocysts exposed to *SmHGPRTases*-dsRNAs. **(A)** Bars representing the mortality percentage of sporocysts exposed to specific dsRNAs and control groups as described in the legend. Above the bars are represented the standard error of the mean of two biological replicates. As the statistical test, Two-way ANOVA with Sidak’s multiple comparisons was used. Black asterisks represent significant differences from untreated control and gray asterisks from unspecific controls. **(B)** Area (μm^2^) of sporocysts exposed to the SmHGPRTases-dsRNAs. Symbols represent a single parasite for each experimental group. Black circles, untreated control; White squares, unspecific control; Black square, parasites exposed to *SmHGPRTase 1*-dsRNA; White triangles, parasites exposed to *SmHGPRTase 2/4/5*-dsRNA; Black triangles, parasites exposed to *SmHGPRTase 3*-dsRNA; White rhombus, combined group. The horizontal black lines represent the median area of the sample population. After verifying the normality using the D’Agostino-Pearson test, significant differences compared to control conditions were analyzed by unpaired *t*-test. **(C–E)** Representative images of the untreated control group **(C)**, sporocysts exposed to *SmHGPRTase 1*-dsRNA **(D)**, and sporocyst from the combined group **(E)** 7 days after dsRNA exposure. White arrows indicate changes in phenotype or dead sporocysts (*p ≤ 0.05; **p ≤ 0.01; ***p ≤ 0.001; ****p < 0.0001).

To assess the role of *SmHGPRTases* in sporocysts viability, knockdown parasites were daily observed. After 4 days of exposure to *SmHGPRTase 1*-dsRNA, a mortality of 8% rate in sporocysts was observed, 62% higher than the rate shown by the untreated control. This value was maintained until the seventh day, when the sporocysts reached a mortality rate of 19%, a value 57% higher than those found in the untreated and unspecific control groups. For sporocysts exposed to *SmHGPRTase 2/4/5*-dsRNA, a mortality rate of 13% after 7 days of exposure presented a significant increase when compared with the nonspecific control. The combined group had the highest sporocysts mortality rate, showing a mortality rate of 12% at third day of exposure to dsRNAs, representing a 75% increase in mortality compared to the two control groups. On the seventh day, this group reached the mortality rate of 22%, being approximately 65% higher than both control groups (Figure 6A).

Assessment of parasite phenotypes after the knockdown of *SmHGPRTase 1, 2/4/5*, and *3* was also performed. The sporocysts area was delimited considering the day in which each gene presented the lowest transcript levels. A significant reduction (8–13%) in the area of sporocysts exposed to *SmHGPRTase 1-* and *2/4/5*-dsRNA was noted when compared to control groups after 4 days of dsRNA exposure. *SmHGPRTase 3* knocked-down sporocysts did not show a significant alteration in their size (Figure 6B). Figures 6C–E show representative images evidencing the higher number of dead sporocysts in each group.

### *SmHGPRTases* knockdown can be involved in schistosomula growth

**Figure 7:**
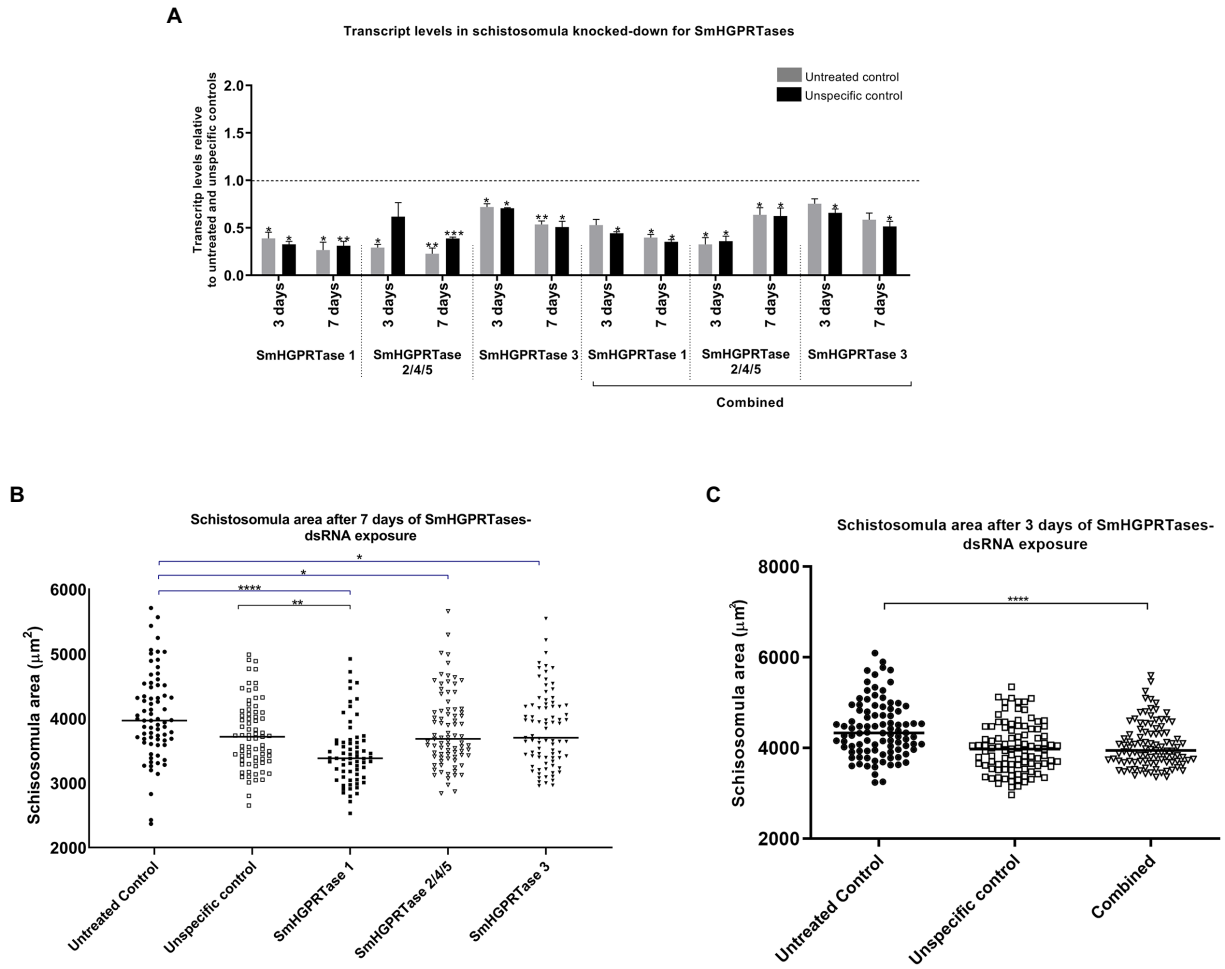
*SmHGPRTases* transcript levels and area of schistosomula after exposure to dsRNAs. **(A)** Bars representing the transcript levels of *SmHGPRTase 1, SmHGPRTase 2/4/5,* and *SmHGPRTase 3* in schistosomula after exposure to the specific dsRNAs separately or in combination (combined) relative to the untreated (gray) or unspecific control (black) after 3 and 7 days. Above the bars are represented the standard error of the mean of three replicates. After verifying the normality using the Shapiro–Wilk test, significant differences compared to control conditions were analyzed by unpaired *t*-test. **(B)** Area (μm^2^) of schistosomula exposed to the *SmHGPRTases-dsRNAs* after 7 days. Symbols represent a single parasite for each experimental group. Black circles, untread control; White squares, unspecific control; Black square, parasites treated with *SmHGPRTase 1*-dsRNA; White triangles, parasites treated with *SmHGPRTase 2/4/5-dsRNA*; Black triangles, parasites treated with *SmHGPRTase 3*-dsRNA. **(C)** Area (μm^2^) of schistosomula exposed to the combined group after 3 days. Symbols represent a single parasite for each experimental group. Black circles, untread control; White squares, unspecific control; White triangles, combined group. The horizontal black lines represent the median area of the sample population. After verifying the normality using the D’Agostino-Pearson test, significant differences compared to control conditions were analyzed by unpaired *t*-test (*p ≤ 0.05; **p ≤ 0.01; ***p ≤ 0.001; ****p < 0.0001).

In the schistosomula stage, transcript levels were optimally reduced during all assessments, resulting in up to ~85% for *SmHGPRTase 1* compared with both controls. Using *SmHGPRTase 2/4/5*-dsRNA the knockdown resulted in 40–71% after 3 days, and 62–78% after 7 days of exposure when compared with the untreated and unspecific controls. For *SmHGPRTase 3*, lower transcript levels were observed after 7 days of dsRNA exposure (50%). When in combination, the *SmHGPRTases* transcript levels only reduced after the third day of dsRNA exposure when reached its maximum knockdown for *SmHGPRTase 2/4/5* (68%). However, for *SmHGPRTase 1* and *SmHGPRTase 3,* the maximum knockdown was detected after seven days, reaching ~63%, and approximately 49%, respectively (Figure 7A; Supplementary Figure S3).

Concerning the measurement of adenosine levels for the schistosomula life stage, no statistically significant difference was observed when the adenosine levels obtained for knocked-down parasites were compared with the unspecific control (Supplementary Figure S4A). On the other hand, when the schistosomula area was delimited considering the day in which each gene presented the lowest transcript levels, it was observed 12% of area reduction for parasites treated with *SmHGPRTase 1*-dsRNA compared to the untreated control group and 8% compared to the unspecific control group. For *SmHGPRTase 2/4/5*-dsRNA, it was observed a reduction of 7% and for *SmHGPRTase 3*-dsRNA a reduction of 8% compared to the untreated control group. The combined group was also evaluated, and a significant reduction in the schistosomula area of 9% was observed when compared to the untreated control (Figures 7B,C). In addition to the size reduction, dark middle regions and round body in parasites from the combined group were noted (Supplementary Figure S5). The mortality rate in schistosomula did not present any significant differences among the groups (data not shown).

### *SmHGPRTase* knockdown in female adult worms decreased adenosine levels and affect their motility *in vitro*

**Figure 8:**
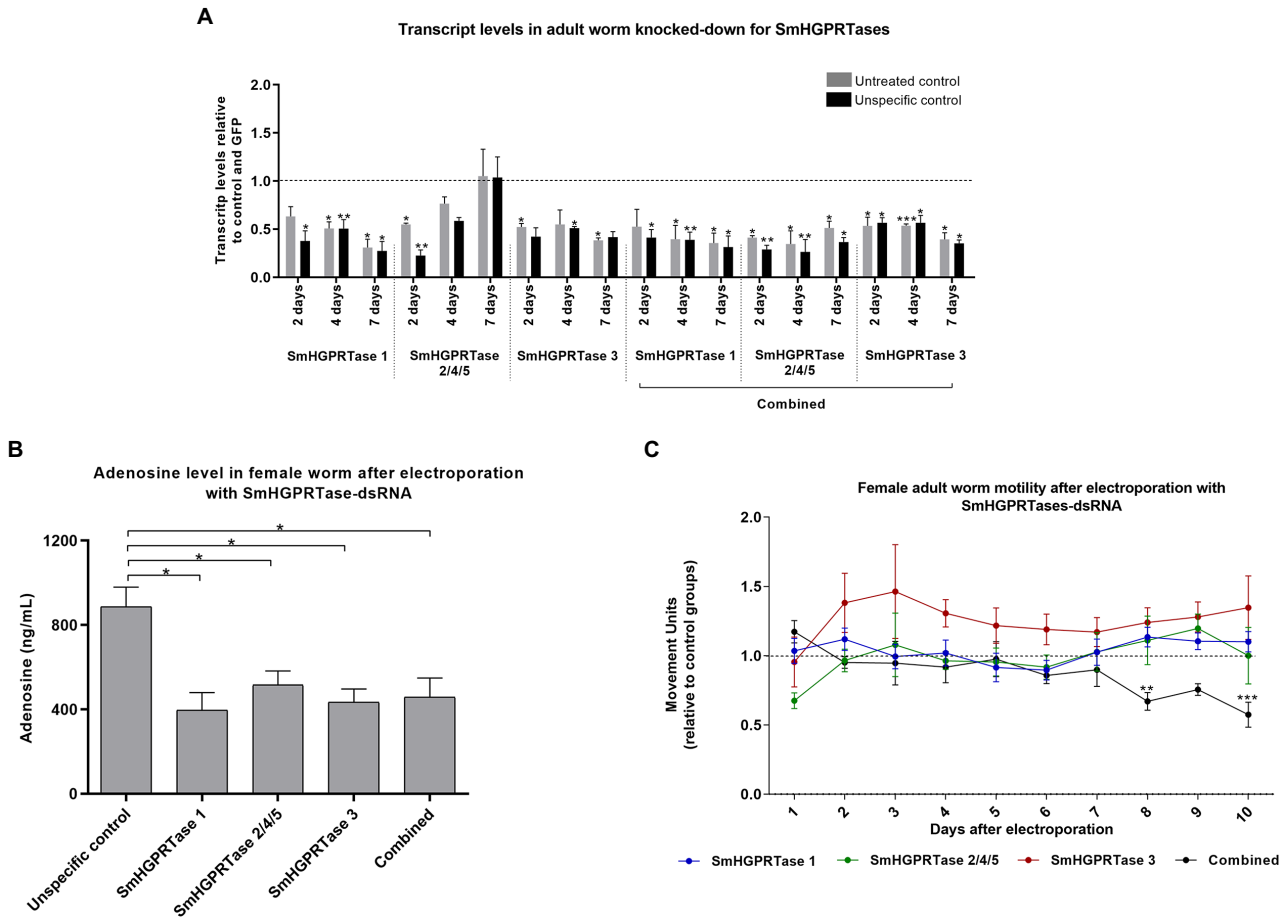
*SmHGPRTases* transcript levels after adult worm electroporation with dsRNAs and knockdown effects in adenosine levels and motility. **(A)** Bars representing the transcript levels of *SmHGPRTase 1, SmHGPRTase 2/4/5,* and *SmHGPRTase 3* in adult worms after electroporation with specific dsRNAs separately or in combination (combined) relative to the untreated (gray) or unspecific control (GFP; black) after 2, 4, and 7 days. Above the bars are represented the standard error of the mean of three replicates. After verifying the normality using the Shapiro–Wilk test, significant differences compared to control conditions were analyzed by unpaired *t*-test. **(B)** Bars representing the adenosine levels in female adult worms in the respective groups: unspecific control, parasites exposed to *SmHGPRTase 1*-dsRNA, *SmHGPRTase 2/4/5*-dsRNA, *SmHGPRTase 3*-dsRNA, and the combined group. Above the bars are represented the standard error of the mean of three replicates. After verifying the normality using the Shapiro–Wilk test, significant differences compared to control conditions were analyzed by unpaired *t*-test. **(C)** Motility of female adult worms exposed to *SmHGPRTases*-dsRNAs. Dots represent the average of the movement units of female adult worms that were electroporated with *SmHGPRTases*-dsRNAs. Blue, parasites exposed to *SmHGPRTase 1*-dsRNA; Green, parasites exposed to *SmHGPRTase 2/4/5*-dsRNA; Red, parasites exposed to SmHGPRTase 3-dsRNA; Black, parasites exposed to the combination of the three *SmHGPRTases*-dsRNAs. The results were normalized according to the movement units of female adult worms from controls (dotted line). Standard errors of the mean of three biological replicates are represented above the dots. Statistical analysis was performed using Two-way ANOVA followed by the Sidak post-test (*p ≤ 0.05; **p ≤ 0.01; ***p ≤ 0.001).

When adult worms were electroporated with the specific dsRNAs, *SmHGPRTase 1* presented the highest knockdown on the seventh days after exposure 70%. On the other hand, *SmHGPRTase 2/4/5* gene presented no transcript reduction on that day and reduced by 46% compared to untreated control group and 78% compared to nonspecific control group 2 days after electroporation. A decrease of 48–60% in the *SmHGPRTase 3* transcript levels was stable during all days the transcript levels were assessed. When the combined group was analyzed, similar results were seen for *SmHGPRTase 1* and *SmHGPRTase 3*, however, for *SmHGPRTase 2* lower transcript levels were detected. On the second, fourth, and seventh days after electroporation, a decrease of 59–72%, 66–74%, and 49–64%, respectively (Figure 8A).

After that, the adenosine levels were checked to certify if they had decreased in adult worms. In female adult worms, the adenosine levels were reduced by approximately 45% for all the *SmHGPRTases* knocked-down parasites compared with the unspecific control (Figure 8B). On the other hand, for adult male worms no statistically significant difference in the adenosine levels was observed (Supplementary Figure S4B).

To analyze changes in the adult worms’ viability after *SmHGPRTase* knockdown, the parasite’s movement was quantified. Female adult worms electroporated with the combined *SmHGPRTases*-dsRNAs showed a significant reduction in their movement on the eighth (34%) and tenth day (43%) compared to the untreated and unspecific controls (Figure 8C). In male adult worms, no significant difference in their movement was observed (Supplementary Figure S6).

### *SmHGPRTases* knockdown interferes in the ovary development and egg maturation in female adult worms*in vivo*

To evaluate the function of *SmHGPRTases* in the infection establishment, schistosomula exposed to the combined *SmHGPRTases*-dsRNAs were used for *in vivo* experiments using a murine model.

**Figure 9:**
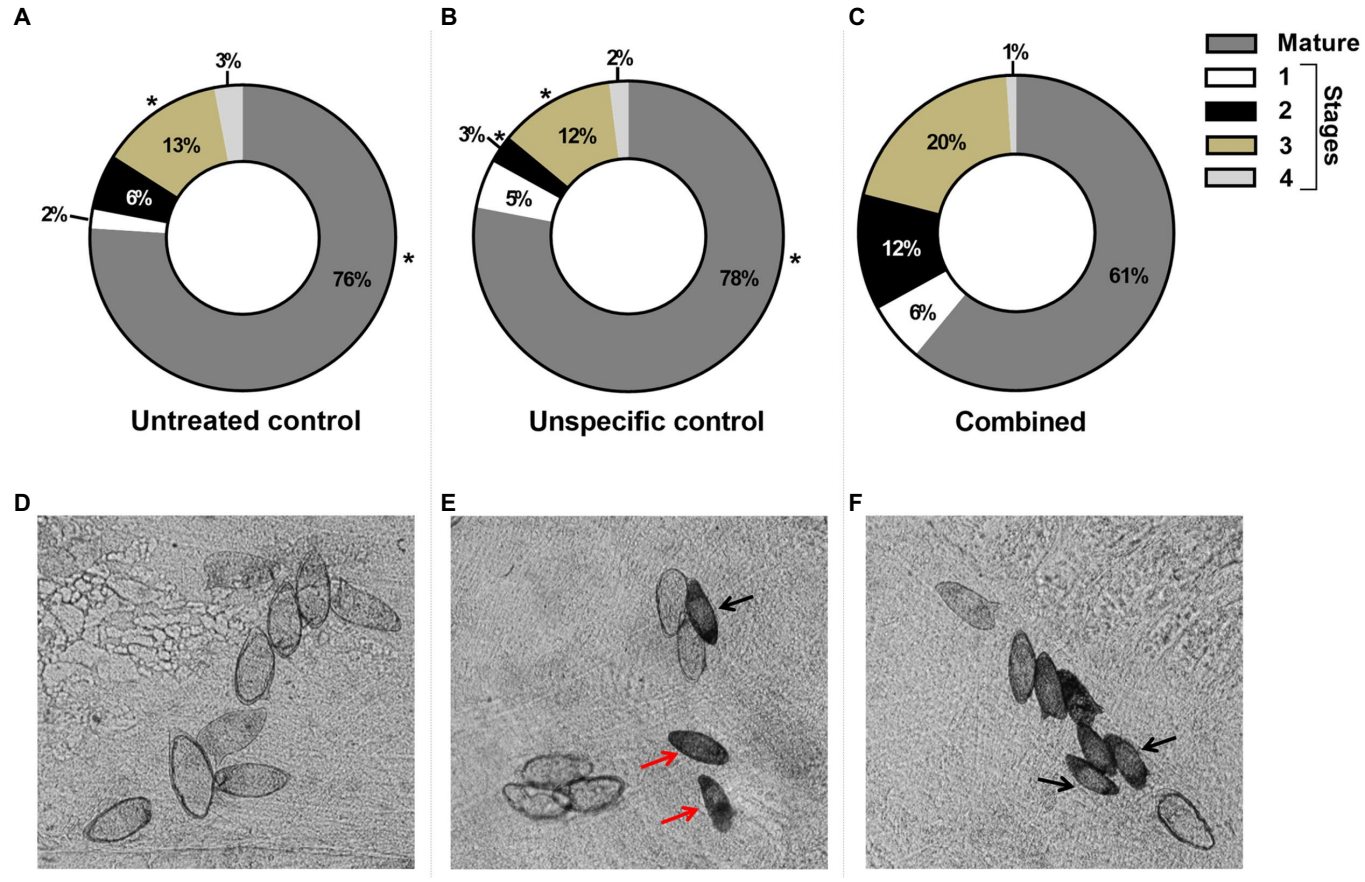
Egg maturation in the ileum of mice infected with schistosomula exposed to the dsRNAs of the five *SmHGPRTases* combined. The percentage of eggs in each maturation stage is represented in the circle section: mature (gray), stage 1 (white), stage 2 (black), stage 3 (beige), and stage 4 (light gray) for the untreated control group (**A**), GFP-dsRNA unspecific control (**B**), and the combined group (**C**). After verifying the normality using the Shapiro–Wilk test, significant differences compared to control conditions were analyzed by unpaired *t*-test (*p ≤ 0.05). (**D–E**) Representative images of the maturation pattern in each experimental group. Black arrow, eggs in stage 2; Red arrow, eggs in stage 3.

After perfusion, the number of recovered adult worms and eggs from the liver and intestine from the experimental groups did not show significant differences when compared to the controls (data not shown). However, the ileum of mice infected with schistosomula exposed to the combined *SmHGPRTases-*dsRNAs presented a significant increase in immature eggs. A significant increase of 8% of eggs in stage 3 was found in mice infected with knocked-down parasites when compared to the controls. Also, an increase (9%) in eggs on stage 2 when compared to the unspecific control was observed. Additionally, a significant reduction of 16% in mature eggs was observed in the combined group (Figures 9A–C). Representative images in Figures 9C–D highlight the differences among eggs found in the mice ileum.

**Figure 10:**
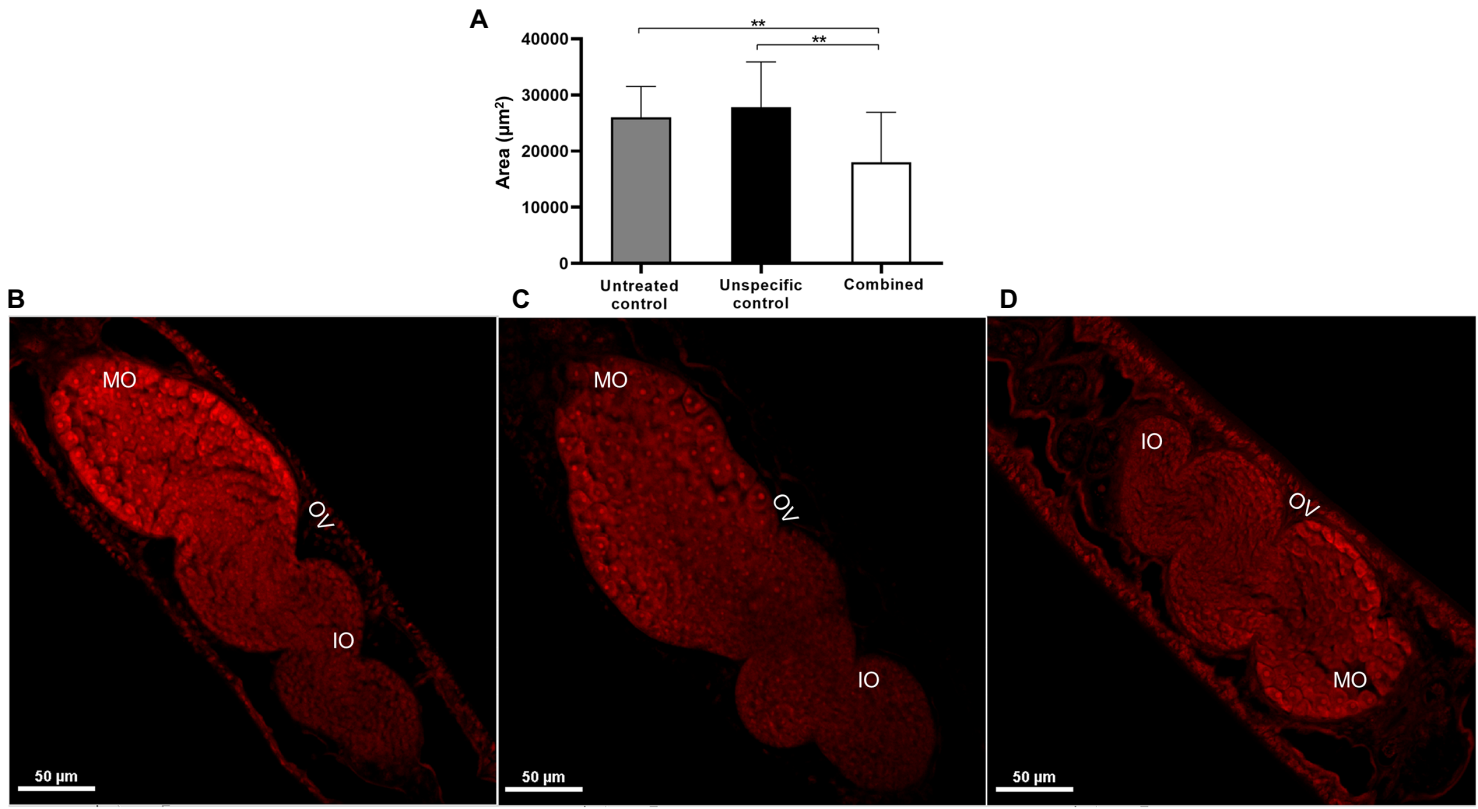
Phenotypic changes in female adult worms recovered from mice infected with *SmHGPRTases* knocked-down schistosomula. **(A)** Bars representing the ovary area of female adult worms from the untreated control (gray), unspecific control (black), and combined *SmHGPRTases* group (white). Above the bars are represented the standard error of the mean of three replicates. After verifying the normality using the Shapiro–Wilk test, significant differences compared to controls were analyzed by unpaired *t*-test (**p ≤ 0.01). **(B–D)** Representative confocal images demonstrating female adult worms recovered from the untreated control **(B)**, unspecific control **(C)**, and the combined *SmHGPRTases* group **(D)**. MO, mature oocyte; IO, immature oocyte; OV, ovary. Scale bars: 50 μm.

Confocal microscopy was performed to verify if the parasite morphology was also altered in association with the high number of immature eggs noted after *SmHGPRTases* knockdown. A significant reduction of 36% in the ovary area was observed in female adult worms recovered from mice infected with schistosomula knocked-down for all *SmHGPRTases* when compared with controls (Figure 10A). Figures 10B–D demonstrates representative confocal images of the structural changes observed in female adult worms due to *SmHGPRTases* knockdown. No alterations were observed in male adult worms’ reproductive systems (data not shown).

## Discussion

Functional studies have demonstrated that Smp38 is important for *S. mansoni* infection establishment in the mammalian host. This protein seems to be involved in the development of reproductive structures, the antioxidant defense, and egg maturation in this parasite (Avelar et al., 2019). In addition, studies have shown that Smp38 is essential in the regulation of miracidia ciliary movement and the early post-embryonic parasite development (Ressurreição et al., 2011a,b). The transcriptome of Smp38 knocked-down schistosomula showed that Smp38 regulates essential processes for the establishment of parasite homeostasis, including genes coding for proteins related to the structural composition of ribosomes, spliceosomes, and cytoskeleton, besides genes involved in the purine and pyrimidine metabolism pathways (Gava et al., 2019).

Analysis of Smp38 protein structure (Kelley et al., 2015) shows approximately 60% similarity with their respective human orthologs. The identity is even greater when only the domain sequences are compared. Accordingly, it would be interesting to explore parasite genes regulated by Smp38 MAPK pathway, that differ from the human orthologs or are parasite-specific, since several studies have proved the essential roles of Smp38 for *S. mansoni* development (Ressurreição et al., 2011a,b; Avelar et al., 2019; Gava et al., 2019). Thus, we sought to understand the biological functions of some genes that are regulated by this kinase in the parasite development, which may be responsible for the phenotypic alterations observed in Smp38 knocked-down parasites, among those genes are the hypoxanthine-guanine phosphoribosyl transferase 1 (*SmHGPRTase 1*) (Gava et al., 2019).

Different from their mammalian hosts, schistosomes lack the *de novo* purine biosynthesis pathway and depend on the salvage pathway for their purine requirements. One of the parasite’s advantages in using the salvage pathway is the fact that the *de novo* biosynthesis requires a large amount of energy to synthesize puric bases. However, it also implies that schistosomes present a great dependence on an external supply of preformed puric bases in order to develop (Miech et al., 1975; Senft and Crabtree, 1983). For this reason, the salvage pathway has been raised as an interesting target for the development of new specific drugs to support schistosomiasis treatment (Ross and Jaffe, 1972; Craig et al., 1988; Yuan et al., 1992), among them, purine analogs inhibit enzymes of *S. mansoni* salvage pathway (Ross and Jaffe, 1972; Miech et al., 1975). Tubercidin is one of those and causes the separation of *S. mansoni* worm pairs, loss of suction capacity of the ventral suction cup, alteration of the muscular activity pattern, and egg-laying inhibition (Ross and Jaffe, 1972). Hypoxanthine-guanine phosphoribosyl transferases (HGPRTases), targets of this study, are essential enzymes in the purine salvage pathway.

Since transcriptomic data are a snapshot of transcription rates, generating an insight of general biological data that requires posterior refinement, thus, we aimed at corroborating previously data generated by transcriptomics approach, using molecules predicted to bind to the Smp38 ATP-binding site to confirm the regulation of *SmHGPRTase* expression. We observed significant changes in the parasite phenotype indicating that they can be promising protein inhibitors, even more by the fact that these kinases are unique members of their families in *S. mansoni* (Rask-Andersen et al., 2014; Moreira et al., 2022). Besides that, we confirmed that Smp38 seems to regulate the expression of *SmHGPRTase 1*. In addition, we investigated whether Smp38 MAPK also regulates the expression of other *SmHGPRTase* family members. We observed that, although *SmHGPRTase 3* was not detected among the differentially expressed genes using the transcriptome data, the Smp38 inhibition seems to negatively regulate the expression of this gene. Analyzing the parasite’s genome organization, we found that the five genes encoding SmHGPRTases are located on chromosome IV, in which *SmHGPRTases 1* and *3* appear to be regulated by Smp38 MAPK, and are proximally located. On the other hand, the genes encoding *SmHGPRTases 2, 4,* and *5,* which the expression does not appear to be regulated by this signaling pathway are also closely distributed evidencing *in tandem* duplications as already described by Wang et al. (2017). The gene duplication analysis revealed that genes derived from proximal duplications diverge less in gene structure and expression levels than the dispersed duplicated genes. The retention of duplicated genes is a mechanism described to contribute to the expansion of gene families associated with pathogenesis and consequent adaptations to parasitism for *S. mansoni* (Wang et al., 2017).

Previous studies showed that female adult worms recovered from mice infected with Smp38 knocked-down schistosomula showed a reduction in the ovary area and male adult worms presented a reduction in the tubercles’ height (Avelar et al., 2019). Our results demonstrated that female adult worms recovered from mice, after 42 days of infection with *SmHGPRTases* knocked-down schistosomula, also presented similar phenotype reaffirming the fact that the Smp38 pathway may be related to the regulation of *SmHGPRTase* expression.

*SmHGPRTases* also show high expression in female adult worms. Previous studies suggest that these genes may be involved in the parasite’s sexual maturation (Romanello et al., 2019). Also, whole-mount *in situ* hybridization experiments show that SmHGPRTase 2 is present along the body of male and female worms, possibly indicating a sexual specialization role for this enzyme (Romanello et al., 2019). Genes that code for SmHGPRTases were also found up-regulated in parasites 28 days after infection in the mammalian host, suggesting a possible role for these enzymes in the synthesis of molecules necessary for egg production (Buddenborg et al., 2019). Our results corroborate these findings. Moreover, female adult worms knocked-down for all *SmHGPRTases* reduced their movement 8 days after dsRNA exposure *in vitro*.

Interestingly, when we checked the available single-cell RNAseq data, we observed that *SmHGPRTses 1* and *3* are more expressed in mature female worm’s reproductive organs, including the cells from early, mature, and late vitellocytes, late female germ cells, and germline stem cell, in comparison with immature females (Wendt et al., 2020). This may explain the fact that egg maturation was altered, and adult worms recovered from mice infected with *SmHGPRTases* knocked-down schistosomula display phenotypic changes in the female reproductive organs.

*In silico* analysis showed that SmHGPRTases present important differences in protein structures and domain sequences when compared to their respective human ortholog. This data reaffirms other studies whereupon schistosomal HGPRTase structure has been compared with the human HGPRTase by circular dichroism. Significant differences were observed in thermostability and in the amino-acid side chains. The schistosomal protein contains 27% α-helix and 30% β-sheet, whereas the human enzyme contains 21% α-helix and 53% β-sheet (Yuan et al., 1993). In addition, the steady-state kinetics mechanism was determined and suggests that the design of a highly specific SmHGPRTase inhibitor, that binds exclusively to the enzyme-purine nucleotide binary complex, may be possible (Yuan et al., 1992). On the other hand, a study using isothermal titration calorimetry to determine kinetic parameters of SmHGPRTases 1, 2, and 3 observed that KM values for schistosomal HGPRTase are very similar to those determined for the human HGPRTase, indicating that SmHGPRTase alone is unlikely to be an efficient therapeutic target (Romanello et al., 2019).

Additionally, our data also show that *SmHGPRTase 1* and *SmHGPRTase 2/4/5* genes exhibited the highest expression levels in the sporocyst stage. In fact, in the snail host, extensive parasite asexual reproduction takes place, and several generations of multiplying sporocysts develop (Maldonado and Acosta Matienzo, 1947) which would demand energy and nucleotides for DNA synthesis, supporting the greater expression of *SmHGPRTases* in this stage. Corroborating these data, the analysis of the *in vivo* parasite transcriptome 3 days post-infection in *Biomphalaria pfeifferi* demonstrated an up-regulation of genes related to the purine salvage and nucleotide biosynthesis pathways (Buddenborg et al., 2019).

Small interfering RNAs (siRNAs) targeting *SmHGPRTase 1* have been used to interrogate this enzyme role in the parasite during the infection in mice. In the study, siRNAs were inoculated in the tail of *S. mansoni* infected mice, resulting in a 27% reduction in worm burden after a 60% reduction in the transcript levels (Pereira et al., 2008). This reduction could reflect the duplicity of functions among the HGPRTases present in the parasite and our results reinforce the importance of knocking down or inhibiting all genes of this family. This mechanism is evident by the higher mortality levels found in sporocysts when all the genes were knocked-down simultaneously. Our results also suggest that *SmHGPRTase 1* may be a major player for the sporocyst viability in regardregard to the other members of the family, since the mortality rate is also high when we knockdown this gene alone. However, it is important to point out that *SmHGPRTase 3* did not present a significant reduction in the transcript levels in sporocysts, consequently no significant phenotypic alterations can be attributed to the latter.

In summary, here we show that the Smp38 MAPK pathway regulates the expression of *SmHGPRTase 1* and *SmHGPRTase 3*. All members of the *SmHGPRTase* family seem to have a functional role in the adenosine uptake. Our results also suggest that SmHGPRTases activity is important for schistosomula development and that SmHGPRTase 1 and SmHGPRTase 2/4/5 are essential for sporocysts viability and development. Phenotypic alterations, for instance: parasites with dark central-region and reduction in female movement *in vitro*; were more evident when all gene family were knocked-down simultaneously, implying an overlap on the SmHGPRTase functions. SmHGPRTases also play a role in the parasite reproduction, demonstrated by the reduced ovary area in female adult worms and the significant increase in the number of immature eggs in the mice ileum. In conclusion, this study is a step forward in the elucidation of parasite biology and the functional roles of SmHGPRTases in *S. mansoni*, reinforcing the importance of these enzymes as therapeutic targets candidates in the parasite.

## Data availability statement

Publicly available datasets were analyzed in this study. This data can be found here: ncbi.nlm.nih.gov/bioproject/, PRJNA611783, SRASRP252217.

## Ethics statement

The animal study was reviewed and approved by Animals’ procedures were approved by the Ethics Commission on Animal Use (CEUA) of the Oswaldo Cruz Foundation under the numbers LW-12/16 and LM-05/18. Experiments were performed under Brazil national guidelines following Law 11794/08.

## Author contributions

MM, SG, and IB contributed to the conception and design of the study. MM contributed with reagents, materials, and analysis tools. IB and NT performed the *in vivo* experiments. IB performed the *in vitro* experiments and modeling analyses. IB and CC-S performed the statistical analyses. SG performed the single-cell analysis. MM, IB, SG, NT, and CC-S wrote the manuscript and contributed to manuscript revision and approved the submitted version. All authors contributed to the article and approved the submitted version.

## Funding

This research was supported by grants from the European Commission’s Seventh Framework Programme for research, under grant agreement no. 602080 (A-ParaDDisE), CAPES PCDD-Programa CAPES/Nottingham University (3661/2014), FAPEMIG (CBB-APQ-0520-13), Conselho Nacional de Desenvolvimento Cientifico e Tecnológico (CNPq) (Fellowship Grant number 302518/2018–5 and 317389/2021–1), Rede de Plataformas Tecnológicas Fiocruz (PDTIS). This study was also financed in part by the Coordenação de Aperfeiçoamento de Pessoal de Nível Superior – Brasil (CAPES) – Finance Code 001.

## Conflict of interest

The authors declare that the research was conducted in the absence of any commercial or financial relationships that could be construed as a potential conflict of interest.

## Publisher’s note

All claims expressed in this article are solely those of the authors and do not necessarily represent those of their affiliated organizations, or those of the publisher, the editors and the reviewers. Any product that may be evaluated in this article, or claim that may be made by its manufacturer, is not guaranteed or endorsed by the publisher.

## Footnotes

^1^ https://parasite.wormbase.org/Schistosoma_mansoni_prjea36577

^2^ https://www.uniprot.org

^3^ https://pfam.xfam.org/

^4^ https://mafft.cbrc.jp/

^5^ http://www.jalview.org

^6^ http://www.sbg.bio.ic.ac.uk/phyre2

^7^ https://www.ncbi.nlm.nih.gov/geo/

^8^ http://primer3.sourceforge.net

^9^ www.graphpad.com

## References

[ref1] Andrade L. F. Nahum L. A. Avelar L. G. A. Silva L. L. Zerlotini A. Ruiz J. C. (2011). Eukaryotic protein kinases (ePKs) of the helminth parasite *Schistosoma mansoni*. BMC Genomics 12:215. doi: 10.1186/1471-2164-12-215, PMID: 21548963 PMC3117856

[ref2] Avelar L. D. G. A. Gava S. G. Neves R. H. Silva M. C. S. Araújo N. Tavares N. C. (2019). Smp38 MAP kinase regulation in *Schistosoma mansoni*: roles in survival, oviposition, and protection against oxidative stress. Front. Immunol. 10:21. doi: 10.3389/fimmu.2019.00021, PMID: 30733716 PMC6353789

[ref3] Bateman A. Martin M. J. Orchard S. Magrane M. Agivetova R. Ahmad S. (2021). UniProt: the universal protein knowledgebase in 2021. Nucleic Acids Res. 49, D480–D489. doi: 10.1093/nar/gkaa1100, PMID: 33237286 PMC7778908

[ref4] Botros S. S. Bennett J. L. (2007). Praziquantel resistance. Expert Opin. Drug Discovery 2, S35–S40. doi: 10.1517/17460441.2.S1.S35 23489031

[ref5] Boyle S. N. Koleske A. J. (2007). Dissecting kinase signaling pathways. Drug Discov. Today 12, 717–724. doi: 10.1016/j.drudis.2007.07.019 17826684

[ref6] Buddenborg S. K. Kamel B. Hanelt B. Bu L. Zhang S. M. Mkoji G. M. (2019). The *in vivo* transcriptome of *Schistosoma mansoni* in the prominent vector species *Biomphalaria pfeifferi* with supporting observations from *Biomphalaria glabrata*. PLoS Neglec. Trop. Dis. 13:e0007013. doi: 10.1371/journal.pntd.0007013, PMID: 31568484 PMC6797213

[ref7] Craig S. P. 3rd McKerrow J. H. Newport G. R. Wang C. C. (1988). Analysis of cDNA encoding the hypoxanthine-guanine phosphoribosyltransferase (HGPRTase) of *Schistosoma mansoni*; a putative target for chemotherapy. Nucleic Acids Res. 16, 7087–7101. doi: 10.1093/nar/16.14.7087, PMID: 3136439 PMC338353

[ref8] Cuesta-Astroz Y. Santos A. Oliveira G. Jensen L. J. (2019). Analysis of predicted host–parasite interactomes reveals commonalities and specificities related to parasitic lifestyle and tissues tropism. Front. Immunol. 10:212. doi: 10.3389/fimmu.2019.00212, PMID: 30815000 PMC6381214

[ref9] Mourão M. Dinguirard N. Franco G. R. Yoshino T. P. (2009). Phenotypic screen of early-developing larvae of the blood fluke, *Schistosoma mansoni*, using RNA interference. PLoS Negl. Trop. Dis. 3:e502. doi: 10.1371/journal.pntd.0000502, PMID: 19668375 PMC2719580

[ref10] Doenhoff M. J. Cioli D. Utzinger J. (2008). Praziquantel: mechanisms of action, resistance and new derivatives for schistosomiasis. Curr. Opin. Infect. Dis. 21, 659–667. doi: 10.1097/QCO.0b013e328318978f, PMID: 18978535

[ref11] Dovey H. F. McKerrow J. H. Wang C. C. (1984). Purine salvage in *Schistosoma mansoni* schistosomules. Mol. Biochem. Parasitol. 11, 157–167. doi: 10.1016/0166-6851(84)90062-8, PMID: 6431283

[ref12] Eglen R. M. Reisine T. (2009). The current status of drug discovery against the human kinome. Assay Drug Dev. Technol. 7, 22–43. doi: 10.1089/adt.2008.164, PMID: 19382888

[ref13] El-Gebali S. Mistry J. Bateman A. Eddy S. R. Luciani A. Potter S. C. (2019). The Pfam protein families database in 2019. Nucleic Acids Res. 47, D427–D432. doi: 10.1093/nar/gky995, PMID: 30357350 PMC6324024

[ref14] Gava S. G. Tavares N. C. Falcone F. H. Oliveira G. Mourão M. M. (2019). Profiling transcriptional regulation and functional roles of *Schistosoma mansoni* c-Jun N-terminal kinase. Front. Genet. 10:1036. doi: 10.3389/fgene.2019.01036, PMID: 31681440 PMC6813216

[ref15] Greenberg R. M. (2013). New approaches for understanding mechanisms of drug resistance in schistosomes. Parasitology 140, 1534–1546. doi: 10.1017/S0031182013000231, PMID: 23552512 PMC3775338

[ref16] Gu Z. Eils R. Schlesner M. (2016). Complex heatmaps reveal patterns and correlations in multidimensional genomic data. Bioinformatics 32, 2847–2849. doi: 10.1093/bioinformatics/btw313, PMID: 27207943

[ref17] Hanks S. K. Quinn A. M. Hunter T. (1988). The protein kinase family: conserved features and deduced phylogeny of the catalytic domains. Science 241, 42–52. doi: 10.1126/science.3291115, PMID: 3291115

[ref18] Howe K. L. Bolt B. J. Shafie M. Kersey P. Berriman M. (2017). WormBase ParaSite − a comprehensive resource for helminth genomics. Mol. Biochem. Parasitol. 215, 2–10. doi: 10.1016/j.molbiopara.2016.11.005, PMID: 27899279 PMC5486357

[ref19] Kaiser M. M. Baszczyňski O. Hocková D. Poštová-Slavětínská L. Dračínský M. Keough D. T. (2017). Acyclic nucleoside phosphonates containing 9-deazahypoxanthine and a five-membered heterocycle as selective inhibitors of plasmodial 6-oxopurine phosphoribosyltransferases. ChemMedChem 12, 1133–1141. doi: 10.1002/cmdc.201700293, PMID: 28628279

[ref20] Katoh K. Rozewicki J. Yamada K. D. (2019). MAFFT online service: multiple sequence alignment, interactive sequence choice and visualization. Brief. Bioinform. 20, 1160–1166. doi: 10.1093/bib/bbx108, PMID: 28968734 PMC6781576

[ref21] Kelley L. A. Mezulis S. Yates C. M. Wass M. N. Sternberg M. J. E. (2015). The Phyre2 web portal for protein modeling, prediction and analysis. Nat. Protoc. 10, 845–858. doi: 10.1038/nprot.2015.053, PMID: 25950237 PMC5298202

[ref22] Keough D. T. Rejman D. Pohl R. Zborníková E. Hocková D. Croll T. (2018). Design of *Plasmodium vivax* hypoxanthine-guanine phosphoribosyltransferase inhibitors as potential antimalarial therapeutics. ACS Chem. Biol. 13, 82–90. doi: 10.1021/acschembio.7b00916, PMID: 29161011

[ref23] Livak K. J. Schmittgen T. D. (2001). Analysis of relative gene expression data using real-time quantitative PCR and the 2−ΔΔCT method. Methods 25, 402–408. doi: 10.1006/meth.2001.1262 11846609

[ref24] Machado-Silva J. R. Lanfredi R. M. Gomes D. C. (1997). Morphological study of adult male worms of *Schistosoma mansoni* Sambon, 1907 by scanning electron microscopy. Mem. Inst. Oswaldo Cruz 92, 647–653. doi: 10.1590/S0074-02761997000500016, PMID: 9566233

[ref25] Maldonado J. F. Acosta Matienzo J. (1947). The development of *Schistosoma mansoni* in the snail intermediate host, *Australorbis glabratus*. P.R. J. Public Health Trop. Med. 22, 331–373. 20264246

[ref26] Marcellino C. Gut J. Lim K. C. Singh R. McKerrow J. Sakanari J. (2012). WormAssay: A novel computer application for whole-plate motion-based screening of macroscopic parasites. PLoS Neglect. Trop. Dis. 6:e1494. doi: 10.1371/journal.pntd.0001494, PMID: 22303493 PMC3269415

[ref27] Mati V. L. T. Melo A. L. (2013). Current applications of oogram methodology in experimental schistosomiasis; fecundity of female *Schistosoma mansoni* and egg release in the intestine of AKR/J mice following immunomodulatory treatment with pentoxifylline. J. Helminthol. 87, 115–124. doi: 10.1017/S0022149X12000144, PMID: 22390937

[ref28] Miech R. P. Senft A. W. Senft D. G. (1975). Pathways of nucleotide metabolism in *Schistosoma mansoni*—VI adenosine phosphorylase. Biochem. Pharmacol. 24, 407–411. doi: 10.1016/0006-2952(75)90226-9, PMID: 1125049

[ref29] Milligan J. N. Jolly E. R. (2011). Cercarial transformation and *in vitro* cultivation of *Schistosoma mansoni* schistosomules. J. Vis. Exp. 54:3191. doi: 10.3791/3191, PMID: 21876520 PMC3217644

[ref30] Moreira B. P. Batista I. C. A. Tavares N. C. Armstrong T. Gava S. G. Torres G. P. (2022). Docking-based virtual screening enables prioritizing protein kinase inhibitors with *in vitro* phenotypic activity against *Schistosoma mansoni*. Front. Cell. Infect. Microbiol. 12:913301. doi: 10.3389/fcimb.2022.913301, PMID: 35865824 PMC9294739

[ref31] Pearce E. J. Sher A. (1987). Mechanisms of immune evasion in schistosomiasis. Contrib. Microbiol. Immunol. 8, 219–232. PMID: 3304833

[ref32] Pellegrino J. Siqueira A. F. (1956). A perfusion technic for recovery of *Schistosoma mansoni* from experimentally infected guinea pigs. Rev. Bras. Malariol. Doencas. Trop. 8, 589–597. 13494879

[ref33] Pereira T. C. Pascoal V. D. B. Marchesini R. B. Maia I. G. Magalhães L. A. Zanotti-Magalhães E. M. (2008). *Schistosoma mansoni*: evaluation of an RNAi-based treatment targeting HGPRTase gene. Exp. Parasitol. 118, 619–623. doi: 10.1016/j.exppara.2007.11.017, PMID: 18237732

[ref34] Pettersen E. F. Goddard T. D. Huang C. C. Couch G. S. Greenblatt D. M. Meng E. C. (2004). UCSF Chimera? A visualization system for exploratory research and analysis. J. Comput. Chem. 25, 1605–1612. doi: 10.1002/jcc.20084, PMID: 15264254

[ref35] R Core Team (2021). A Language and Environment for Statistical Computing. Vienna: R Foundation for Statistical Computing

[ref36] Rask-Andersen M. Zhang J. Fabbro D. Schiöth H. B. (2014). Advances in kinase targeting: current clinical use and clinical trials. Trends Pharmacol. Sci. 35, 604–620. doi: 10.1016/j.tips.2014.09.007 25312588

[ref37] Ressurreição M. Rollinson D. Emery A. M. Walker A. J. (2011a). A role for p38 MAPK in the regulation of ciliary motion in a eukaryote. BMC Cell Biol. 12:6. doi: 10.1186/1471-2121-12-6, PMID: 21269498 PMC3040701

[ref38] Ressurreição M. Rollinson D. Emery A. M. Walker A. J. (2011b). A role for p38 mitogen-activated protein kinase in early post-embryonic development of *Schistosoma mansoni*. Mol. Biochem. Parasitol. 180, 51–55. doi: 10.1016/j.molbiopara.2011.07.002, PMID: 21787807

[ref39] Romanello L. Zeraik A. E. de Freitas Fernandes A. Torini J. R. Bird L. E. Nettleship J. E. (2019). *In vitro* and *in vivo* characterization of the multiple isoforms of *Schistosoma mansoni* hypoxanthine-guanine phosphoribosyltransferases. Mol. Biochem. Parasitol. 229, 24–34. doi: 10.1016/j.molbiopara.2019.02.005, PMID: 30772423

[ref40] Ross A. F. Jaffe J. J. (1972). Effects of tubercidin and its ribonucleotides on various metabolic pathways in *Schistosoma mansoni*. Biochem. Pharmacol. 21, 3059–3069. doi: 10.1016/0006-2952(72)90198-0, PMID: 4346386

[ref41] Satija R. Farrell J. A. Gennert D. Schier A. F. Regev A. (2015). Spatial reconstruction of single-cell gene expression data. Nat. Biotechnol. 33, 495–502. doi: 10.1038/nbt.3192, PMID: 25867923 PMC4430369

[ref42] Senft A. W. Crabtree G. W. (1983). Purine metabolism in the schistosomes: potential targets for chemotherapy. Pharmacol. Ther. 20, 341–356. doi: 10.1016/0163-7258(83)90031-1, PMID: 6412258

[ref43] Tavares N. C. Gava S. G. Torres G. P. de Paiva C. Ê. S. Moreira B. P. Lunkes F. M. N. (2020). *Schistosoma mansoni* FES tyrosine kinase involvement in the mammalian schistosomiasis outcome and miracidia infection capability in *Biomphalaria glabrata*. Front. Microbiol. 11:963. doi: 10.3389/fmicb.2020.00963, PMID: 32595609 PMC7300192

[ref44] Tavares N. Mourão M. (2021). Parasitemia evaluation in mice infected with *Schistosoma mansoni*. Bio Protoc. 11:e4017. doi: 10.21769/BioProtoc.4017, PMID: 34150924 PMC8187114

[ref45] Vidhya V. M. Ponnuraj K. (2021). Structure-based virtual screening and computational study towards identification of novel inhibitors of hypoxanthine-guanine phosphoribosyltransferase of *Trypanosoma cruzi*. J. Cell. Biochem. 122, 1701–1714. doi: 10.1002/jcb.30122, PMID: 34346095

[ref46] Wang S. Zhu X. Cai X. (2017). Gene duplication analysis reveals no ancient whole genome duplication but extensive small-scale duplications during genome evolution and adaptation of *Schistosoma mansoni*. Front. Cell. Infect. Microbiol. 7:412. doi: 10.3389/fcimb.2017.00412, PMID: 28983471 PMC5613093

[ref47] Waterhouse A. M. Procter J. B. Martin D. M. Clamp M. Barton G. J. (2009). Jalview version 2--a multiple sequence alignment editor and analysis workbench. Bioinformatics 25, 1189–1191. doi: 10.1093/bioinformatics/btp033, PMID: 19151095 PMC2672624

[ref48] Wendt G. Zhao L. Chen R. Liu C. O’Donoghue A. J. Caffrey C. R. (2020). A single-cell RNA-seq atlas of *Schistosoma mansoni* identifies a key regulator of blood feeding. Science 369, 1644–1649. doi: 10.1126/science.abb7709, PMID: 32973030 PMC7875187

[ref49] World Health Organization (2018). Schistosomiasis and soiltransmitted helminthiases: numbers of people treated in 2018. Wkly. Epidemiol. Rec., 445–452.

[ref50] World Health Organization (2021). Schistosomiasis - Situation and trends. Available at: https://www.who.int/data/gho/data/themes/topics/schistosomiasis (Accessed September 21, 2022).

[ref51] Yuan L. Craig S. P. 3rd McKerrow J. H. Wang C. C. (1992). Steady-state kinetics of the schistosomal hypoxanthine-guanine phosphoribosyltransferase. Biochemistry 31, 806–810. doi: 10.1021/bi00118a024, PMID: 1731938

[ref52] Yuan L. Wu C. S. C. Craig S. P. 3rd Liu A. F. Wang C. C. (1993). Comparing the human and schistosomal hypoxanthine-guanine phosphoribosyltransferases by circular dichroism. Biochim. Biophys. Acta Protein Struct. Mol. Enzymol. 1162, 10–16. doi: 10.1016/0167-4838(93)90121-7, PMID: 8448172

