# Supplementary material for: Hypoxanthine guanine phosphoribosyl transferases SmHGPRTases functional roles in Schistosoma mansoni
Source: Front Microbiol. 2022 Dec 12;13:1064218. doi: 10.3389/fmicb.2022.1064218 (PMC9791060; doi:10.3389/fmicb.2022.1064218)
Supplement: Supplementary file 1 [file Data_Sheet_1.PDF]

Supplementary table S1 - Specific primers used in this study to carry out the synthesis of dsRNA, RT-qPCR and amplified fragment size (lowercase are the T7 promoter sequences and in capital letters the sequences of the specific primers for each gene).

Target	Gene ID	Forward	Reverse	Amplicon size
**dsRNA primers**				
**SmHGPRTase**	Sm_103560	taatacgactcactatagggAATGTCCGTTGAGTTTGTTCG	taatacgactcactatagggTTCATTAATCACGCAAATATGG	349 bp
	Sm_148820/ Smp_312580/ Smp_332640	taatacgactcactatagggGAGGCTTCAAATTCGCTTCC	taatacgactcactatagggAACTTGTGCCAGTGTCAACC	250 bp
	Sm_168500	taatacgactcactatagggGCCTTCATACAGGAAGAATTTTG	taatacgactcactatagggATTGAGGTTCATGCCCTACG	358 bp
**qPCR primers**				
**SmHGPRTase**	Sm_103560	TGTGTCCTCAAAGGTGGATTTA	CGGACATTGGTAGGACGATAC	94 bp
	Sm_148820/ Smp_312580/ Smp_332640	TGATGATAGTTATGAAGGATATTCAGC	TATCCAAACGACTCCGAACC	113 bp
	Sm_168500	AGGCGGGTTCAAGTTTGC	CCAAACTCACCTCGTATGTGC	122 bp
**Unspecific control dsRNA primers**				
**GFP**	pCRII-GFP	taatacgactcactatagggTCTTCAAGTCCGCCATG	taatacgactcactatagggTGCTCAGGTAGTGGTTGTC	360 bp
**Housekeeping primers**				
**Sm_COXI**	Smp_900000	TACGGTTGGTGGTGTCACAG	ACGGCCATCACCATACTAGC	152 bp
**Sm_Arp10**	Smp_093230	GAAGGCTTGGAAGGATTCTG	CAACTGCCAACTCTCGGATA	137 bp
**Sm_FAD**	Smp_089880	TACGGTTTCTCTTCCCAACC	CATGGACCTGCTGCATTTAC	72 bp
