# Supplementary material for: Hypoxanthine guanine phosphoribosyl transferases SmHGPRTases functional roles in Schistosoma mansoni
Source: Front Microbiol. 2022 Dec 12;13:1064218. doi: 10.3389/fmicb.2022.1064218 (PMC9791060; doi:10.3389/fmicb.2022.1064218)
Supplement: Supplementary file 2 [file Data_Sheet_2.PDF]

Supplementary Table S2. Percent identity of phosphoribosyltransferase domains among HGPRTase proteins found in *S. mansoni* and in humans.

**Domain position** *Schistosoma mansoni*	**Domain position human**	**% identity among domain phosphoribosyltransferase**
**SmHGPRTase 1**<br>**Smp_103560**<br>Position: 58-220	**HGPRTase**<br>Position: 36-196	50,61
**SmHGPRTase 2**<br>**Smp_148820**<br>Position: 43-203		35,23
**SmHGPRTase 3**<br>**Smp_168500**<br>Position: 35-201		40,49
**SmHGPRTase 4/5**<br>**Smp_312580**<br>**Smp_332640**<br>Position: 35-201		36,87
