# Supplementary material for: Hypoxanthine guanine phosphoribosyl transferases SmHGPRTases functional roles in Schistosoma mansoni
Source: Front Microbiol. 2022 Dec 12;13:1064218. doi: 10.3389/fmicb.2022.1064218 (PMC9791060; doi:10.3389/fmicb.2022.1064218)
Supplement: Supplementary file 3 [file Data_Sheet_3.pdf]

Supplementary Material

Supplementary Figures

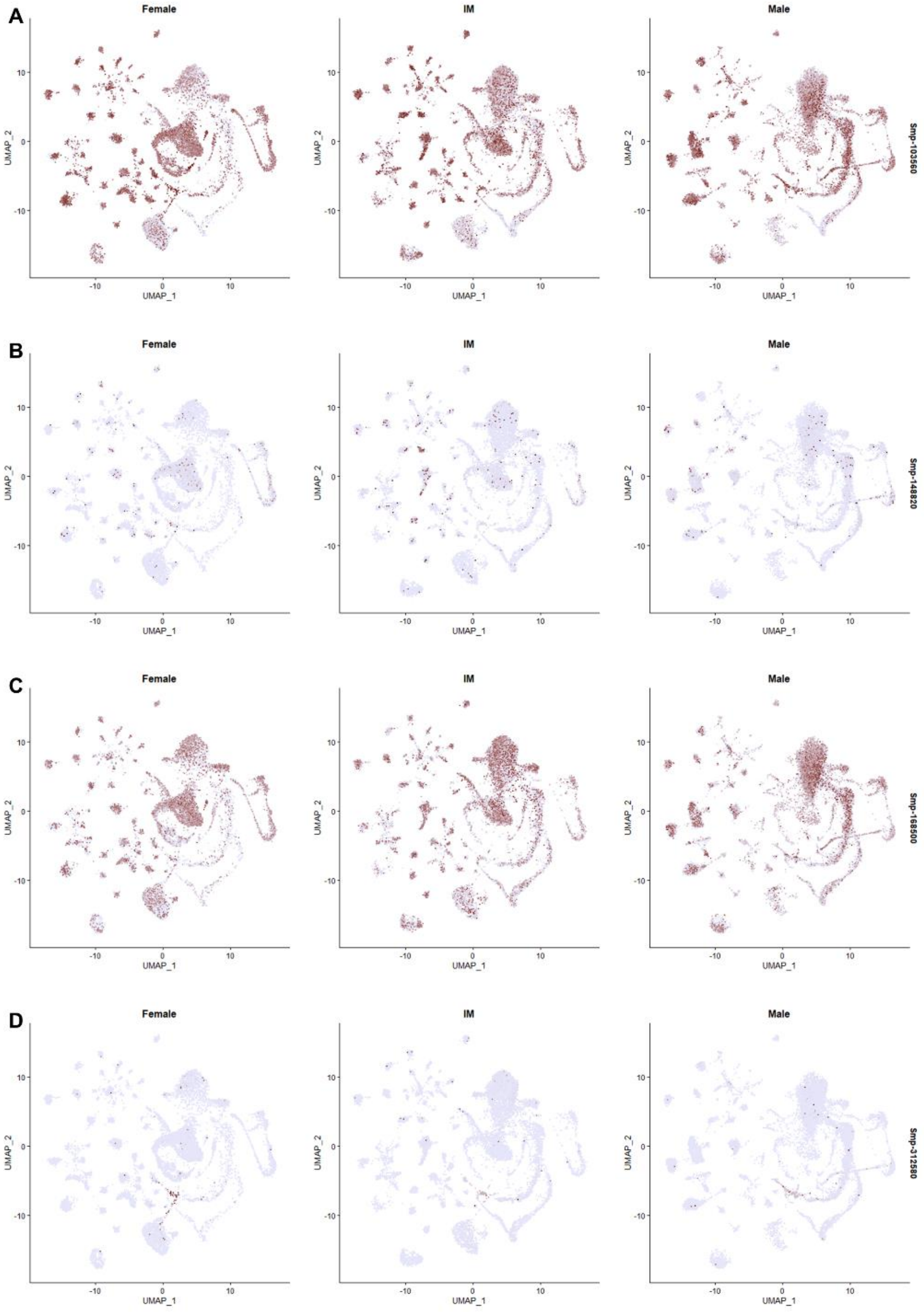

Supplementary Figure S1: SmHGPRTAses expression profiles in different cell clusters identified in *Schistosoma mansoni* adult female, immature female, and male worms. UMAP projection graphs with scRNAseq expression profile in females, immature females (IM) and males of *S. mansoni* from SmHGPRTAses 1 (A), 2 (B), 3 (C), and 4 (D). Expression values were normalized and represented by colors (gray = not expressed, red = expressed).

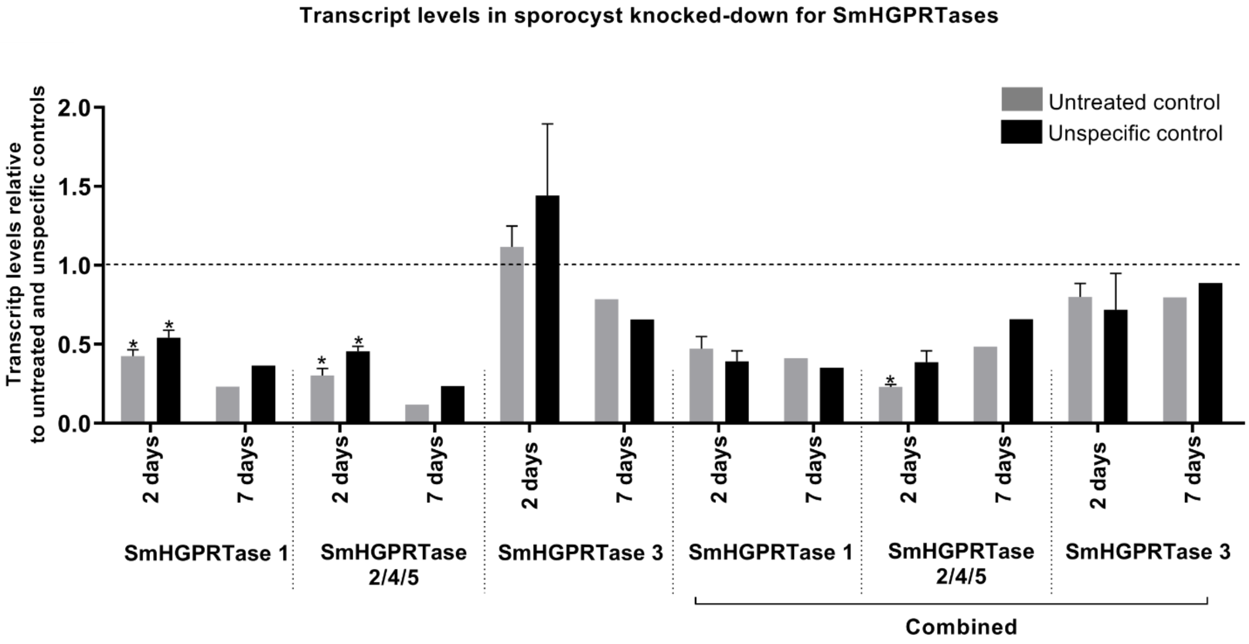

Supplementary Figure S2: *SmHGPRTases* transcript levels after sporocysts exposure to dsRNAs. Bars representing the transcript levels of *SmHGPRTase 1, SmHGPRTase 2/4/5,* and *SmHGPRTase 3* in sporocysts after exposure to the specific dsRNAs separately or in combination (combined) relative to the untreated (grey) or unspecific control (black) after 2 and 7 days. Above the 2-day bars are represented the standard error of the mean of three replicates. The dashed line represents the normalized values in the controls. After verifying the normality using the Shapiro-Wilk test, significant differences compared to control conditions were analyzed by unpaired t-test (* $p \leq 0.05$).

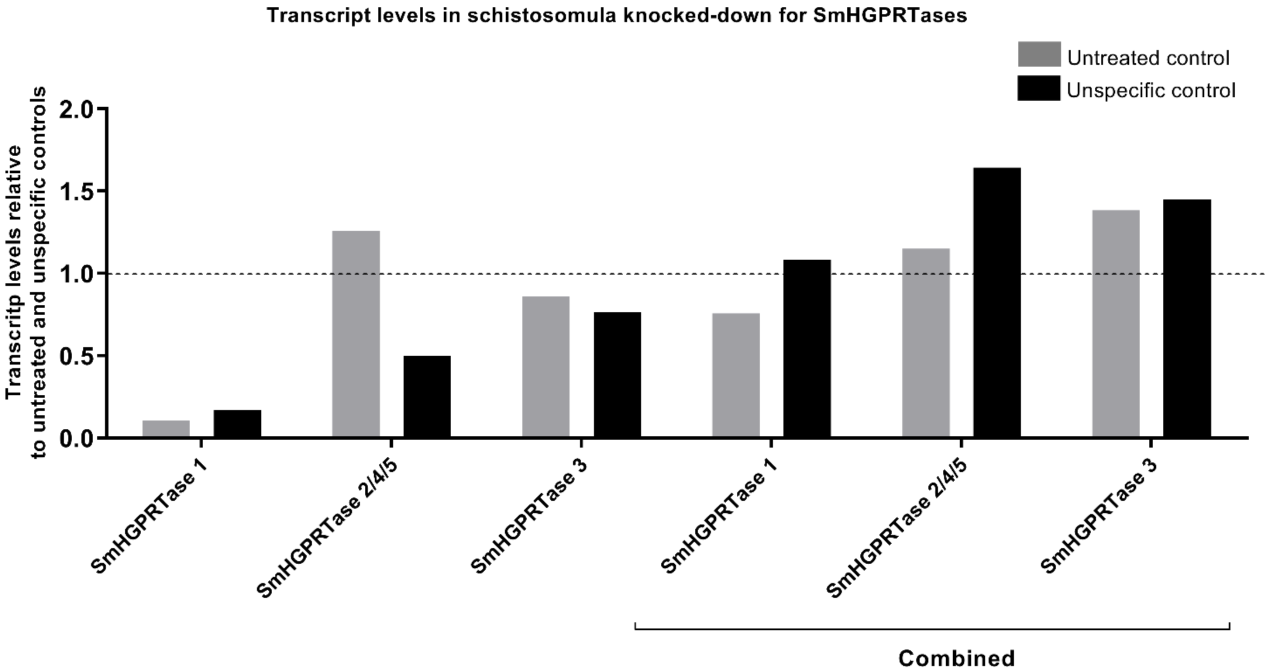

Supplementary Figure S3: SmHGPRTases transcript levels and area of schistosomula after exposure to dsRNAs. Bars representing the transcript levels of *SmHGPRTase 1*, *SmHGPRTase 2/4/5*, and *SmHGPRTase 3* in schistosomula after exposure to the specific dsRNAs separately or in combination (combined) relative to the untreated (grey) or unspecific control (black) after 2 days.

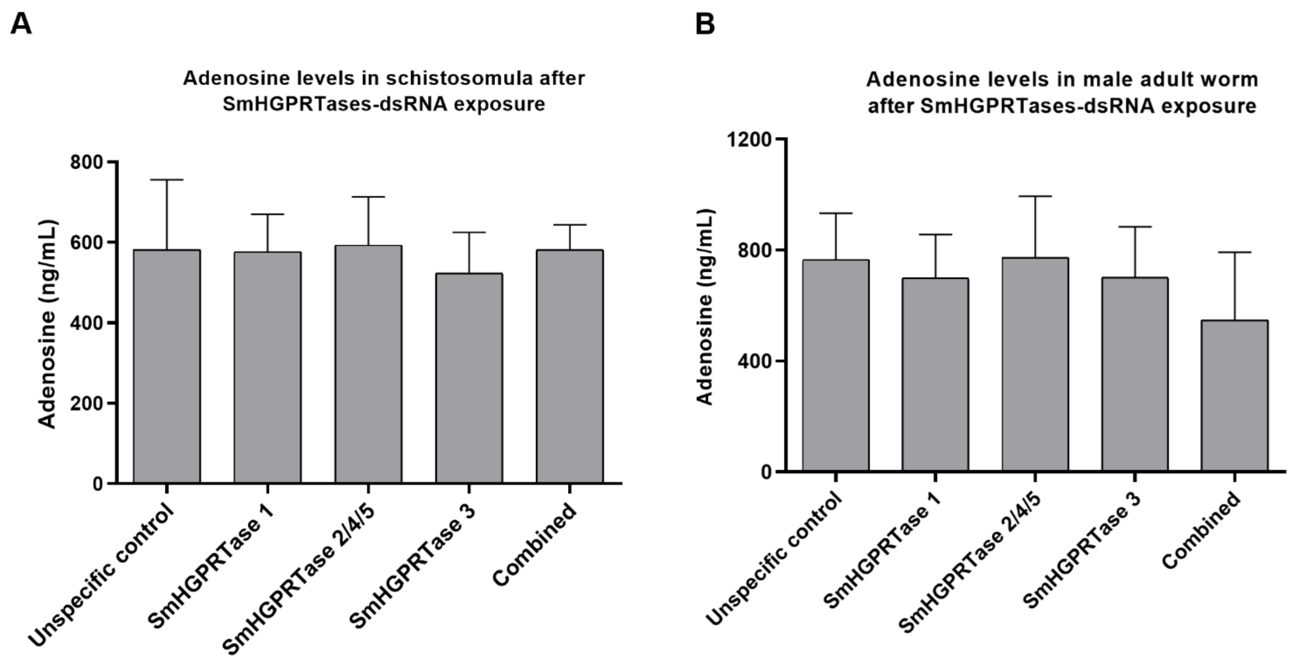

Supplementary Figure S4: Knockdown effects in adenosine levels after parasites exposure to dsRNAs. Bars representing the adenosine levels in schistosomula (A) and in male adult worms (B) in the respective groups: unspecific control, parasites exposed to *SmHGPRTase 1*-dsRNA, *SmHGPRTase 2/4/5*-dsRNA, *SmHGPRTase 3*-dsRNA, and the combined group. Above the bars are represented the standard error of the mean of three replicates.

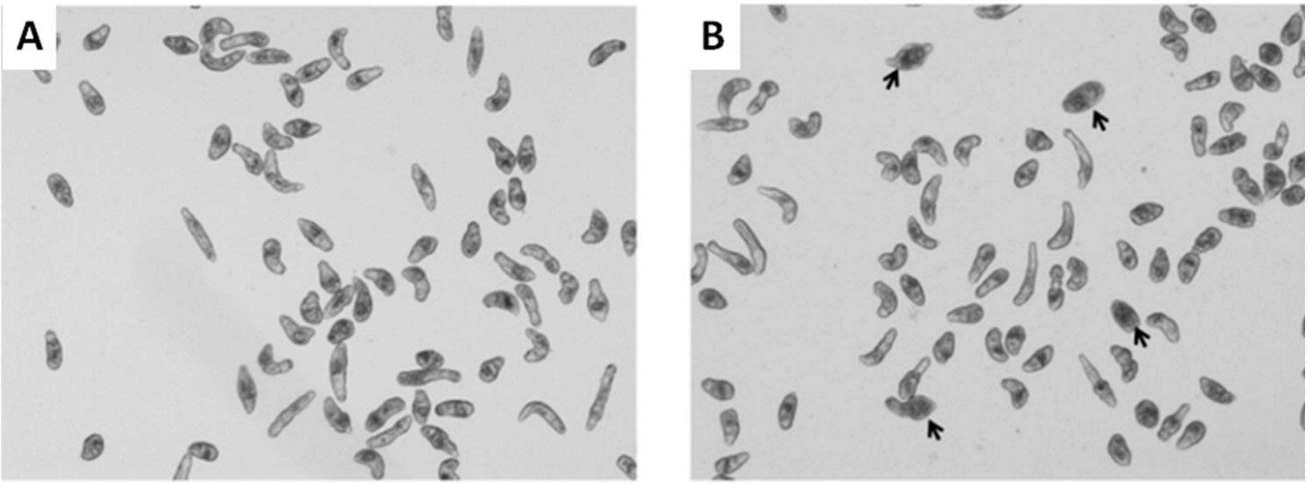

Supplementary Figure S5: Representative images of schistosomula exposed to SmHGPRTases-dsRNA. Untreated control (A) and schistosomula exposed to *SmHGPRTases*-dsRNA (B). Black arrows indicate changes in phenotype or dead schistosomula.

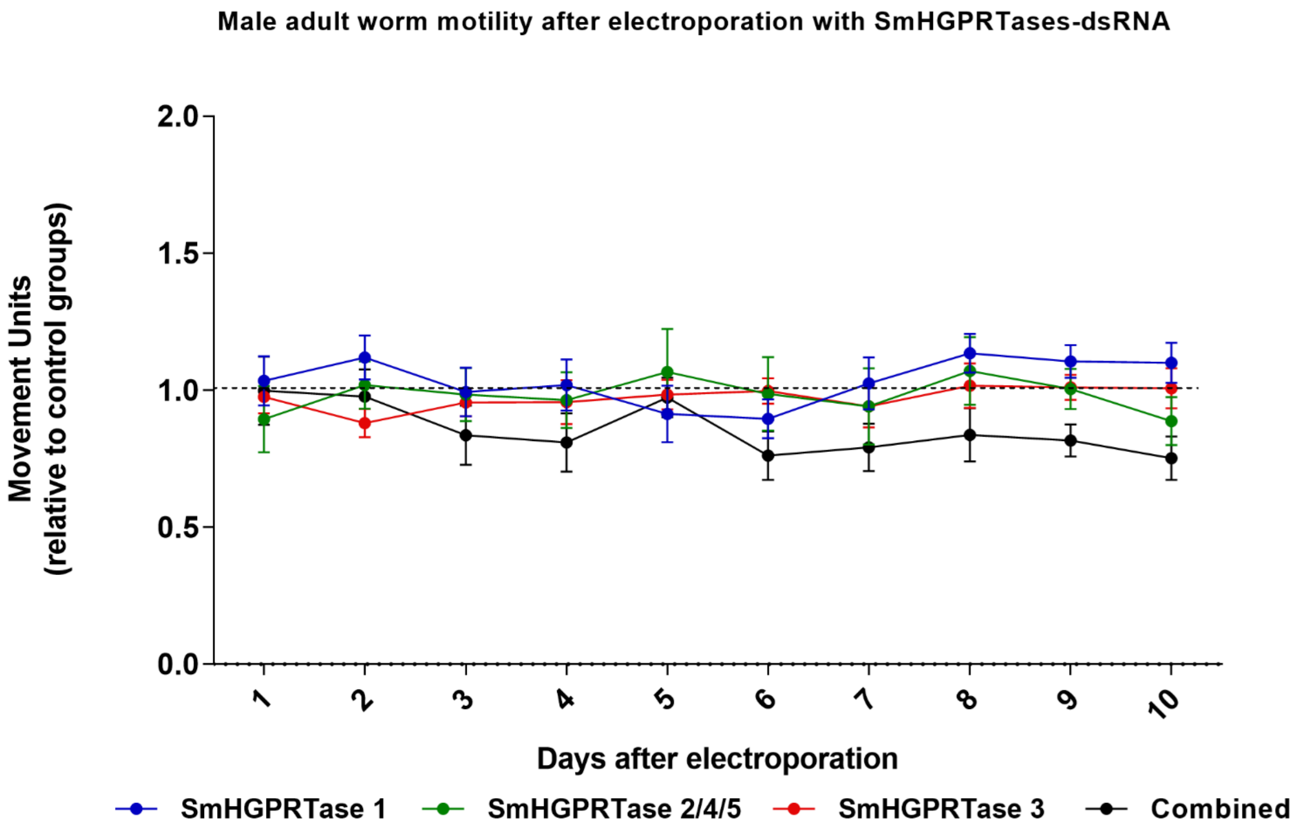

Supplementary Figure S6: Adult male worm's motility after electroporation with SmHGPRTases-dsRNA. Dots represent the average of the movement units of male adult worms that were electroporated with *SmHGPRTases*-dsRNAs. Blue: parasites exposed to *SmHGPRTase 1*-dsRNA. Green: parasites exposed to *SmHGPRTase 2/4/5*-dsRNA. Red: parasites exposed to SmHGPRTase 3-dsRNA. Black: parasites exposed to the combination of the three *SmHGPRTases*-dsRNAs. The results were normalized according to the movement units of adult worms from control groups (dotted line).
